# Thermodynamically Reinforced Dual‐Interface 1D/3D Tin‐Lead Perovskite Heterostructure for Stable All‐Perovskite Tandem Solar Cells

**DOI:** 10.1002/advs.202517732

**Published:** 2025-12-30

**Authors:** Hui Li, Zhen Liu, Bohong Chang, Yutong Wu, Ruiyang Yin

**Affiliations:** ^1^ Key Laboratory for Liquid‐Solid Structural Evolution and Processing of Materials Ministry of Education School of Materials Science and Engineering Shandong University Jinan 250061 P. R. China; ^2^ School of Materials Science and Engineering Peking University Beijing 100871 P. R. China

**Keywords:** 1D/3D perovskite heterostructure, dual‐interface, operational stability, Sn‐Pb perovskites, π‐π stacking

## Abstract

Tin‐lead (Sn‐Pb) perovskite solar cells (PSCs) remain fundamentally constrained by the intrinsic instability of Sn^2+^ oxidation and uncontrollable crystallization, critically limiting their operational reliability in tandem architectures. Herein, this study strategically introduces a π‐conjugated ammonium salt, 4‐aminobenzamidine dihydrochloride, which in situ directs the formation of a dual‐interface one‐dimensional/three‐dimensional (1D/3D) perovskite heterostructure, thereby re‐engineering the Sn‐Pb perovskite lattice toward enhanced thermodynamic stability. The self‐assembled 1D perovskitoid with intermolecular π‐π stacking acts as nucleation‐directing templates to relieve tensile strain and intra‐/intergranular disorder. Simultaneously, the contiguous 1D perovskitoid interphases encapsulating the 3D bulk fortify the vulnerable Sn‐I octahedral framework, effectively obstructing oxidative and ion‐migration pathways. This dual stabilization strategy endows the Sn‐Pb PSCs with unprecedented structural resilience, achieving not only a high power conversion efficiency (PCE) of 22.23% but also a T_98_ operational lifetime beyond 1000 hours. Building upon this enhanced structural robustness, the derived 2‐terminal (2T) all‐perovskite tandem devices deliver a PCE of 28.50% and sustain a T_90_ lifetime of 600 hours, underscoring the central role of lattice stabilization in advancing all‐perovskite tandem photovoltaics.

## Graphical abstract

A π‐conjugated diammonium additive is employed to engineer a dual‐interface 1D/3D perovskite heterostructure. π‐π stacking 1D perovskitoids  act as nucleation templates to relieve tensile strain and intra‐/intergranular disorder and then encapsulate the 3D bulk to fortify the Sn‐I octahedral framework. The all‐perovskite tandem devices based 1D/3D Sn‐Pb heterostructure achieved an efficiency of 28.50% and a T_90_ operational lifetime of 600 hours.

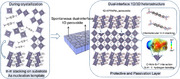

## 1 Introduction

Developing tandem solar cells (TSCs) represents a promising approach to reduce thermalization losses and broaden the usable range of the solar spectrum, thereby increasing the efficiency limit of single‐junction perovskite solar cells (PSCs).^[^ ^1^ ^]^ Leveraging the substantial absorption coefficient and constantly adjustable bandgap, all‐perovskite TSCs offer the advantages of superior efficiency, lightweight design, cheap cost and scalable manufacturing process. Currently, the primary challenge faced in all‐perovskite TSCs is maximizing the efficiency and stability of narrow‐bandgap Sn‐Pb PSCs. On the one hand, inherent structure instability originated from Sn^2+^ oxidation generates deep‐level structural defects, particularly concentrated at the surfaces, thereby aggravating nonradiative recombination losses and accelerating the device degradation.^[^ ^2^, ^3^, ^4^ ^]^ On the other hand, the disordered nucleation and rapid crystallization of Sn‐Pb perovskites lead to inferior film quality, characterized by excessive grain boundaries and non‐uniform surface coverage, which not only significantly limit carrier diffusion lengths but also create defect‐rich regions prone to moisture and oxygen ingress, ion migration, and Sn^2+^ oxidation, collectively compromising the intrinsic and environmental stability of perovskites.^[^ ^5^, ^6^, ^7^, ^8^ ^]^ As a consequence, compared with Pb‐based PSCs, mixed Sn‐Pb counterparts suffer from the more compounded limitations arising from both bulk and interfacial instability.

Currently, the popular surface treatments for perovskite layers have demonstrated effective chemical and field‐effect passivation at the top surface for high performance PSCs, whereas typically offer limited control over the bulk crystallization dynamics of perovskites.^[^ ^9^, ^10^, ^11^, ^12^, ^13^ ^]^ Moreover, at the buried interface, persistent deep‐level defects, such as lead‐halide heterogeneities and sub‐micron structural disorders, promote detrimental interfacial reactions and nonradiative losses.^[^ ^14^, ^15^ ^]^ Attempts to chemically passivate these buried defects often introduce interfacial agents prior to perovskite deposition; however, these agents are prone to being washed away or altered during subsequent spin‐coating steps, thereby undermining their effectiveness and complicating fabrication protocols^[^ ^16^, ^17^ ^]^ Therefore, despite progress in top‐surface engineering, achieving reliable and scalable control over the buried interface remains a substantial challenge.

Encouragingly, the novel in situ dual‐interface optimization while preparing the perovskite films are anticipated to simultaneously achieve both crystallization and interface modulation of Sn‐Pb PSCs. Recently, Wang et al. developed an ionic liquid as precursor additive to realize the in situ defect passivation at both the top and bottom interfaces, thus enabling a certified efficiency of 24.84% and excellent stability for Pb‐based PSCs.^[^ ^17^ ^]^ This in situ dual‐interface passivation approach overcomes the shortcoming of complex and time‐consuming post‐treatment process, showing great promise in the industrialization of PSCs. However, the incorporation of small molecular additives might adversely affect long‐term photothermal stability due to their volatility and weak binding affinity. The emergent low‐dimensional (LD) perovskites offer a more robust alternative, as they not only interact with the dangling bonds on three‐dimensional (3D) perovskites but also stiffen their structural stability. This synergistic reinforcement is particularly advantageous for addressing the inherent instability of Sn‐Pb perovskites under external stresses.

In this work, a structurally stable dual‐interface 1D/3D Sn‐Pb perovskite heterostructure was in situ constructed by introducing the π‐conjugated diammonium salts, 4‐aminobenzamidine dihydrochloride (APFACl), thereby addressing both bulk and interfacial instabilities. The self‐assembled 1D perovskitoid, featuring intermolecular π‐π stacking, functions as an oriented nucleation template, mitigating the lattice strain and crystallization disorder, while simultaneously encapsulating the 3D bulk perovskite to reinforce the fragile Sn‐I framework and suppress Sn^2^⁺ oxidation and ion migration pathways. Accordingly, the engineering 1D/3D Sn‐Pb PSCs deliver a champion PCE of 22.23% along with an exceptional operational stability (T_98_ > 1000 hours), representing one of the most stable Sn‐Pb PSCs reported to date. Importantly, when integrated into 2‐terminal all‐perovskite tandem devices, the structurally stabilized absorbers enable a PCE of 28.50% with a T_90_ lifetime of 600 hours.

## 2 Results and Discussion

**Figure 1:**
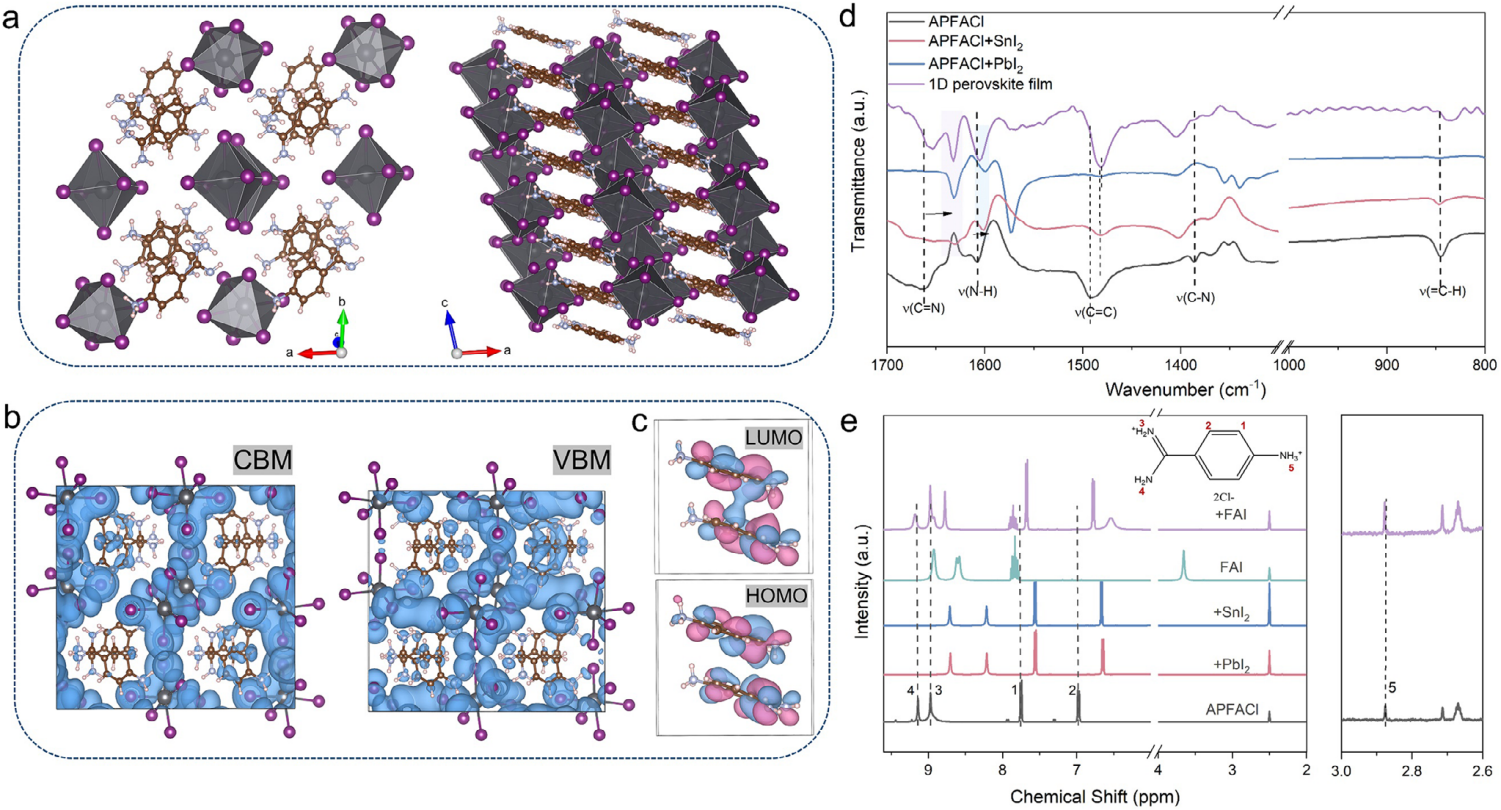
π‐π stacking within 1D perovskitoid. a) Structure illustration of 1D APFAPbI_4_ perovskitoid. b) Charge distribution of CBM and VBM of 1D perovskitoid. c) LUMO and HOMO of adjacent organic moieties. d) FTIR spectra of APFACl, PbI_2_/SnI_2_‐APFACl and 1D APFABI_4_ perovskitoid film. e) ^1^H liquid‐state NMR spectra of APFACl, PbI_2_/SnI_2_‐APFACl, FAI and FAI‐APFACl.

Asymmetric diammonium, known as 4‐aminobenzamidine dihydrochloride (APFACl) was introduced as a perovskite precursor additive to construct the LD/3D perovskite heterostructure. Yellow needle‐like LD perovskite single crystals were synthesized by APFACl and PbO: SnO (1:1) in an aqueous hydriodate iodide and hypophosphorous acid solution (details available in the Experimental sections). **Figures** **1**a and S1 (Supporting Information) shows the analytic crystal structure of APFABI_4_ (B = Sn, Pb) determined by single X‐ray crystallographic data. The crystal structure is classified with the monoclinic P2_1_/c space groups (a = 10.6129 Å, b = 19.2941 Å, c = 8.1854 Å, α = 90°, β = 99.576 °, γ = 90°, Table S1, Supporting Information), exhibiting the 1D linear arrangement along edge‐sharing [PbI_6_]4^−^ octahedron orientation in which organic cations occupy the rectangular A‐site cavities and realize orderly intermolecular π‐π stacking. A wide‐bandgap of 2.19 eV for 1D perovskitoid film is determined from the UV–vis absorption spectra (Figure S2, Supporting Information). Figure 1b shows the calculated conduct band minimum (CBM)‐ and valence band maximum (VBM)‐associated charge distribution of 1D perovskitoid, in which the VBM and CBM are located at I atom and benzene ring, respectively, proving that organic moieties participate in the charge transport process. As shown in Figure 1c, the highest and lowest occupied molecular orbital (HOMO/LUMO) calculation of organic moieties show that the HOMO and LUMO are related with π bonding orbital and π* antibonding orbital for APFA^2+^ spacer cations, respectively. The thick LUMO overlap confirms the π‐π stacking of APFA^2+^ spacer cations in the 1D perovskitoid. Therefore, the presence of the π‐π stacking interaction in the 1D perovskitoid is expected to facilitate the establishment of carrier transport channels, thereby not impeding the charge extraction at the interfaces.

As illustrated in Figure 1d, Fourier transform infrared spectroscopy (FTIR) shows that after mixing with PbI_2_ or SnI_2_, C═N stretching vibration peak (1664 cm^−1^) of APFACl molecules shifts toward a lower wavenumber, which is attributed to the Lewis acid‐base coordination between the electron‐rich C═N groups and Pb^2+^/Sn^2+^ ions. The N‐H bending (1605 cm^−1^) and C‐N stretching vibration peaks (1386 cm^−1^) shift toward higher wavenumbers, which are ascribed to hydrogen bonding and/or ionic interaction between amine/amidine groups and I^−^ ions. Moreover, the shift toward lower wavenumber of the C═C stretching (1492 cm^−1^) and = C‐H (845 cm^−1^) bending vibration peak of benzene ring is related to the electrostatic interactions between the π‐conjugated structure and Pb^2+^/Sn^2+^ ions. Especially C = C stretching and the = C‐H bending vibration peak of benzene ring in 1D APFABI_4_ perovskitoid films exhibit more pronounced peak shifts compared with mechanical mixing with PbI_2_ or SnI_2_. This is mainly attributed to π‐π stacking between adjacent APFA^2+^ spacer cations in the 1D perovskitoid. Next, we conduct the ^1^H liquid nuclear magnetic resonance (NMR) spectroscopy, as shown in Figure 1e. Upon mixing with PbI_2_ or SnI_2_, ‐NH_2_ and = NH_2_^+^ resonance peaks located at δ = 9.14 and 8.97 ppm (4 and 3) in APFACl both exhibit upfield chemical shifts, which is ascribed to the interaction between electron‐rich C═N groups and Pb^2+^/Sn^2+^ ions. After mixing with FAI, the = NH_2_ (4) and ‐NH_3_^+^ (5) peaks present downfield chemical shifts, related to the hydrogen bonding between APFA^2+^ and I^−^ ions. The higher upfield chemical shifts (2 and 1) for APFACl mixed with PbI_2_ or SnI_2_ than FAI are likely related to interactions between the benzene ring and metal ions.^[^ ^18^ ^]^

**Figure 2:**
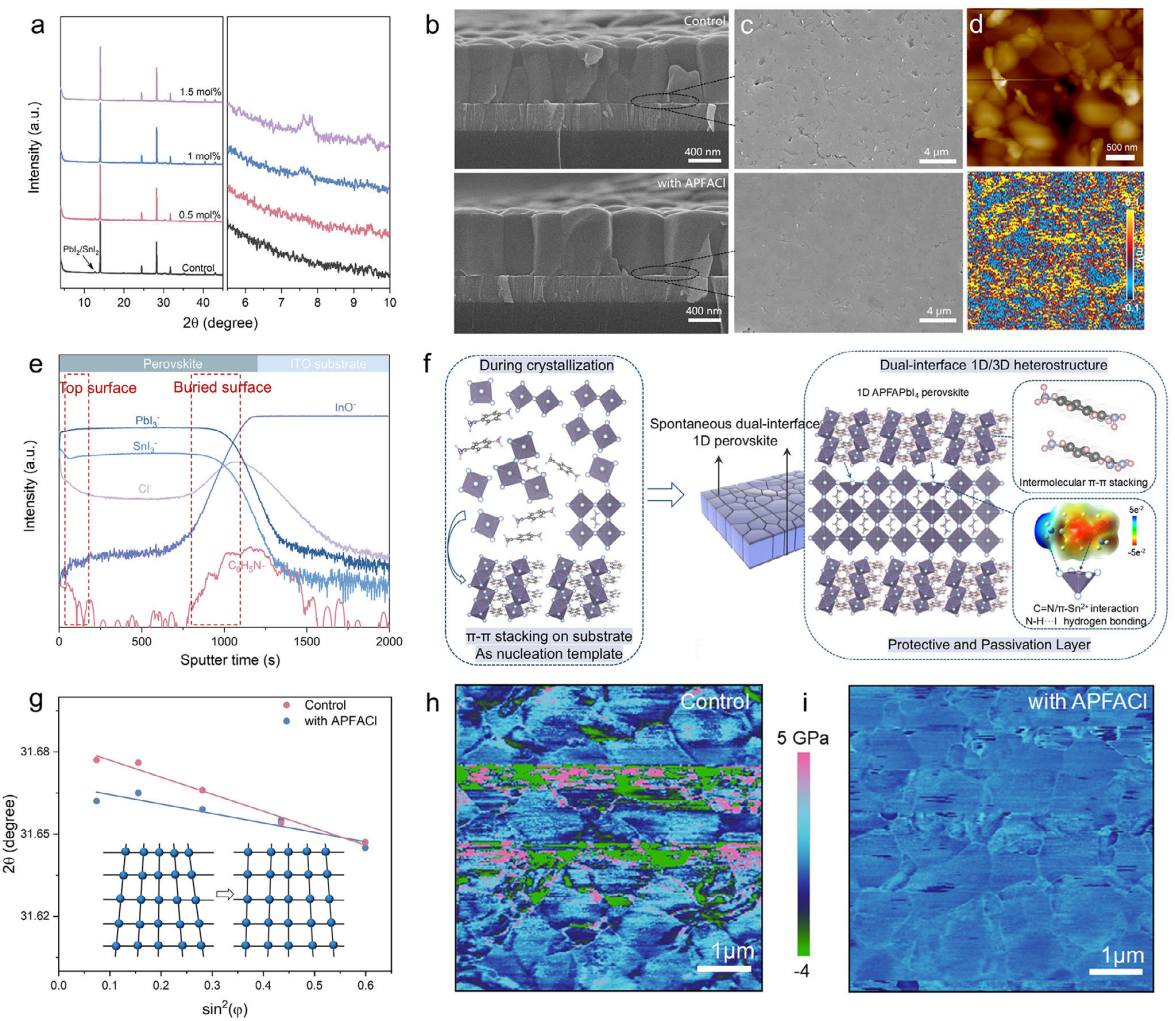
Dual‐interface 1D/3D heterostructure characterization. a) XRD patterns of perovskite films. SEM images of perovskite films of b) top surface, and c) buried surface of control (top) and APFACl‐treated films (bottom). d) AFM images and PTIR images of APFACl‐treated film detected by wavenumber of 1200 cm^−1^. e) TOF‐SIMS profiles of APFACl‐treated perovskite films coated on ITO substrate. f) Schematic diagram illustrating the roles of 1D perovskitoid during and after crystallization. (f) Linear fit of 2θ‐sin^2^(*φ*). PFQNM‐AFM of h) control and i) APFACl‐treated films.

We then investigate the formation mechanism of the 1D/3D heterostructure and elucidate its pivotal role in modulating the crystallization dynamics of Sn‐Pb perovskites. **Figure** **2**a shows X‐ray diffraction (XRD) spectra of perovskite films (with the composition of FA_0.7_MA_0.3_Pb_0.5_Sn_0.5_I_3_, with bandgap of 1.26 eV, Figure S3, Supporting Information) in which the diffraction peaks observed at 14.01°, 24.46°, 28.24°, and 31.70° are assigned to the (110), (202), (220), and (310) crystallographic planes of the 3D perovskite phase, respectively. With increasing APFACl content, the intensity ratio of the (110) to (202) peaks progressively increases, indicating a pronounced preferential orientation along the vertical (out‐of‐plane) direction. Concurrently, the peak at 12.70°, characteristic of residual PbI_2_/SnI_2_, gradually diminishes and a new diffraction peak emerges at approximately 8°, corresponding to the (100) plane of the 1D perovskitoid, confirming the formation of the 1D/3D Sn‐Pb perovskite heterostructure.

Top‐view scanning electron microscopy (SEM) images reveal that PbI_2_/SnI_2_ residues are preferentially segregated at the grain boundaries of control film (Figure S4, Supporting Information). In contrast, the APFACl‐treated film exhibits the formation of needle‐like flake structures at grain boundaries and surfaces, corresponding to the 1D perovskitoid generated via the in situ reaction between PbI_2_/SnI_2_ and APFACl. These low‐dimensional (LD) structures are hypothesized to not only physically encapsulate the grain boundaries against moisture and oxygen ingress, but also chemically passivate deep‐level trap states by neutralizing undercoordinated ions and dangling bonds.^[^ ^19^ ^]^ Moreover, the statistical analysis of grain size by Nano measurement shows an increase in the average grain size of perovskite film (from 490 to 650 nm), suggesting a reduction in inter‐aggregate grain boundaries.^[^ ^20^ ^]^ Meanwhile, cross‐sectional SEM images in Figure 2b further reveal the monolithic grains in the APFACl‐treated film, manifesting the reduction in intra‐aggregate grain boundaries.

Atomic force microscopy‐infrared spectroscopy (AFM‐IR) was employed to investigate the nanoscale distribution of 1D perovskitoid within the 3D perovskite film. Photo‐thermal induced resonance (PTIR) images were obtained through measuring the thermal expansion caused by the absorption of infrared light at chemical bonding‐specific wavelengths using AFM. As shown in Figure 2d, under excitation with IR light at 1200 cm^−1^, corresponding to the aromatic fingerprint region (C‐H bending vibration), strong signals were detected at grain boundaries in APFACl‐treated films and spatially correlates with the flake‐like features observed in AFM images, indicating that 1D perovskitoid is preferentially self‐assembled at grain boundaries via π‐π stacking. Then time‐of‐flight secondary ion mass spectrometry (TOF‐SIMS) was employed to track the vertical distribution of 1D perovskitoid (Figure 2e). The C_6_H_5_N^−^ signal as the marker for the 1D perovskitoid, is mainly distributed at the buried interface, with fewer signals detected at the top surface across the perovskite film. Grazing‐incidence XRD (GIXRD) patterns collected at a 0.3° incident angle further confirmed that the characteristic peaks of 1D perovskitoid signatures appear at both top and buried surfaces of APFACl‐treated films (Figure S5, Supporting Information). Collectively, these results demonstrate that 1D perovskitoid with intermolecular π‐π stacking is predominantly distributed at the surfaces, serving as a crystallization template and protective layer.

To elucidate the mechanism of crystallization modulation, dynamic light scattering (DLS) was performed on the perovskite precursor solutions (Figure S6, Supporting Information). Upon the APFACl addition, large colloidal particles (>1 µm) appeared, likely corresponding to pre‐nucleation clusters formed by the coordination of APFA^2+^ with PbI_2_/SnI_2_. The schematic illustration presented in Figure 2f depicts the underlying mechanism by which the 1D perovskitoid improves the crystallization and stability of Sn‐Pb perovskite. During the crystallization, APFA^2+^ preferentially interacts with PbI_2_/SnI_2_ to generate 1D perovskitoid in the precursor solution. The π‐π stacked 1D perovskitoid tends to sediment onto the substrate, acting as nucleation templates for the subsequent 3D perovskite crystallization.^[^ ^21^, ^22^, ^23^ ^]^ After film formation, the 1D perovskitoid extends along both the top and buried interfaces, in situ wrapping the 3D perovskite grains and providing full‐scale passivation and protection. Structurally, the edge‐sharing [BX_6_]^4−^ octahedra in the 1D framework stabilizes the 5s^2^ (Sn^2+^) and 6s^2^ (Pb^2+^) lone‐pair orbitals, thereby enhancing the lattice robustness. Moreover, electrostatic potential (ESP) mapping of APFA^2+^ reveals an electron‐rich benzene ring and electron‐deficient amino terminus. The aromatic core enhances the electron cloud density around Sn^2+^, raising the oxidation barrier, while the amine/amidine groups form hydrogen bonds interacting with I^−^ ions, collectively stabilizing the Sn‐I octahedral and suppressing degradation pathways.^[^ ^24^ ^]^

The presence of residual tensile strain in perovskite films, arising from the thermal expansion and lattice mismatch between the perovskite and substrate, is widely recognized to compromise the structural stability and aggregate nonradiative recombination. Acting as crystallization‐directing templates, the flexible 1D structure accommodates lattice stress during the cooling process, promoting the preferred orientation and strain‐relieved 1D/3D perovskite films with enhanced intrinsic structure stability. To quantify this, GIXRD with 2θ‐sin^2^φ analysis was conducted (Figure S7, Supporting Information).^[^ ^25^ ^]^ The (310) peak near 31.7° shifted to lower angles with increasing tilt angle (Ψ from 10° to 50°), indicating in‐plane lattice expansion. Linear fitting of 2θ versus sin^2^φ revealed a negative slope, confirming tensile strain. The APFACl‐treated film shows a reduced slope from −0.062 to −0.034, indicating the released tensile strain. Complementarily, Williamson‐Hall (W‐H) analysis indicates a reduction in microstrain (ε) from 3.24×10^−4^ to 2.42×10^−4^ (Figure S8, Supporting Information). Furthermore, As shown in Figure 2g,h, PeakForce Quantitative Nanomechanics (PFQNM) AFM mappings reveal the markedly reduced Young's modulus fluctuations in the APFACl‐treated film (2.245 to 0.218 GPa), suggesting improved mechanical uniformity and strain relaxation.^[^ ^26^, ^27^ ^]^ Building upon the above results, we elucidate that the incorporation of 1D perovskitoids directs the crystallization dynamics of Sn‐Pb perovskite, thereby mitigating intra‐ and inter‐granular disorders and relieving residual tensile strain.

**Figure 3:**
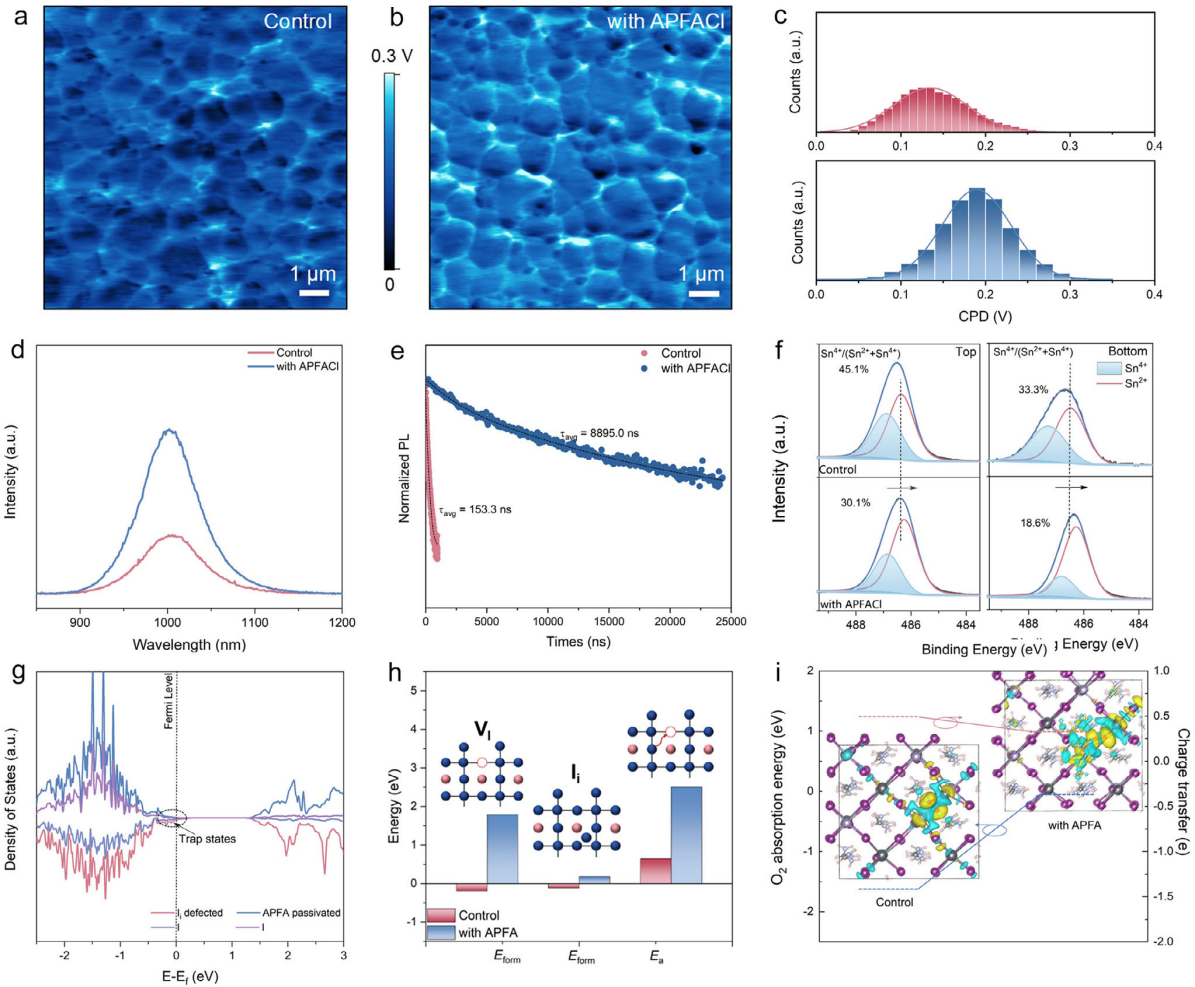
Charge carrier dynamics. KPFM images of a) control and b) APFACl‐treated films. c) Contact potential distribution (CPD) of control (top) and APFACl‐treated films (bottom). d) PL spectra. e) TRPL spectra of perovskite film. f) Sn 3d XPS spectra of top and bottom surface of perovskite films. g) TDOS and PDOS of perovskite: with I_i_ defect and APFA‐passivated perovskite. h) Defect formation energy (*E*_f_) of I‐related defects (V_I_ and I_i_), and active energy of I^−^ ion migration (*E*_a_). i) Geometry‐optimized structures of oxygen‐absorbed V_I_‐perovskite and charge transfer from Sn to O.

Subsequently, we investigate the influence of the 1D perovskitoid functioning as an effective passivation layer. As shown in **Figure** **3**a,b, Kelvin probe force microscopy (KPFM) was performed to elucidate the surface chemical/electrical characteristics. Surface potential fluctuations in perovskite films are associated with local charge variations caused by more reactive defects. The corresponding contact potential difference (CPD) in Figure 3c shows that the APFACl‐treated film exhibits more pronounced potential fluctuations compared with control film, suggesting a reduction in reactive surface defects, as the variations in CPD are associated with localized charge accumulations at defect sites.^[^ ^28^, ^29^ ^]^ Complementary photoluminescence (PL) spectra indicate that the APFACl‐treated films present the improved PL intensities, indicating reduced nonradiative recombination (Figure 3d). Time‐resolved photoluminescence (TRPL) spectra in Figure 3e were well fitted by a biexponential function, where the fast component (*τ*_1_) is attributed to interfacial or trap‐assisted recombination, while the slow component (*τ*_2_) corresponds to bulk carrier recombination (the fitting parameters is shown in Table S2).^[^ ^30^ ^]^ The concurrent enhancement of both τ_1_ and τ_2_ signifies the effective suppression of nonradiative recombination pathways within both the bulk and interfacial regions of the Sn‐Pb perovskite film. Furthermore, the average carrier lifetime (*τ*_avg_) increased markedly from 153.3 ns for the control sample to 8895.0 ns for the APFACl‐treated film (fitting parameters are provided in Table S2, Supporting Information), further confirming the substantial reduction of nonradiative losses.

X‐ray photoelectron spectroscopy (XPS) measurements were performed to study the passivation mechanism associated with chemical interactions (Figure 3f; Figure S9, Supporting Information). Sn 3d and Pb 4f XPS peaks of top and bottom surfaces both shift to the lower binding energy, indicating the higher electron cloud density around the metal ions of perovskite. As shown in Figure 3f, the detrimental Sn^4+^ contents of both top and bottom surface are greatly reduced, thus ameliorating the nonradiative recombination at the contact interface. Moreover, the shift of I 3d XPS to higher binding energies is related to hydrogen bonding or/and electrostatic interaction between I^−^ ions and the amine/amidine groups of APFACl, which facilitates I^−^ anions fixation and I‐related defects passivation.

Density functional theory (DFT) simulations were further performed to elucidate the passivation mechanism based on these interactions. The total and projected density of states (TDOS and PDOS, Figure 3g) reveals that upon APFA adsorption, defect states emerge near the valence band (VB) edge in the presence of I_i_ defects are nearly eliminated, indicating a substantial reduction in trap‐related electronic states.^[^ ^31^ ^]^ Figure 3h elucidates that formation energies of I‐related defect including iodide vacancies (V_I_) and I_i_, are greatly improved following the APFA chemisorption. Moreover, the active energy (*E*_a_) of I^−^ ion migration rises from 0.65 eV to 2.51 eV, indicating a strong suppression of I^−^ ion migration. The formation of I_2_ caused by I^−^ ion migration under illumination can oxidize surface Sn^2+^ to Sn^4+^, thus distorting the Sn‐I framework and accelerating the perovskite degradation.^[^ ^32^, ^33^ ^]^ Tracked by UV–vis absorption spectra obtained by exposing chlorobenzene‐submersed films to one‐sun illumination, a much slower I_2_ release is observed in APFACl‐treated films (Figure S10, Supporting Information), directly proving the improved light stability of perovskite film.^[^ ^34^ ^]^ Then we performed the time‐of‐flight secondary ion mass spectrometry (ToF‐SIMs) of the aged Sn‐Pb PSCs (Figure S11, Supporting Information). For the control devices, evident I^−^ ion migration toward the Ag electrode is accompanied by the diffusion of Ag‐related species into the C_60_ layer, indicative of ion migration process during the aging process. In contrast, such degradation pathways are substantially alleviated in the APFACl‐modified devices.^[^ ^35^, ^36^ ^]^

Beyond halide defects, the oxidative degradation pathway involving superoxide (O_2_^−^) formation is also mitigated. The superoxide species formed by oxygen molecules preferentially absorb on the undercoordinated Sn^2+^ ions, and capture electrons from Sn^2+^ to trigger Sn^2+^ oxidation and subsequent lattice breakdown.^[^ ^35^, ^37^ ^]^ As illustrated in Figure 3i, APFA adsorption raises the formation energy of superoxide species from −1.54 to −0.096 eV, while the charge transfer (*E*_c_) from Sn to O, derived from charge density difference and Bader analysis, is reduced from 0.52 to 0.36 e, indicating a significantly enhanced resistance to oxygen‐induced degradation.^[35]^ As shown in Figure S12 (Supporting Information), the Sn XPS spectra after air exposure reveal that the Sn^4+^/(Sn^4+^+Sn^2+^) ratio reaches 59.2%, which is substantially higher than that of the APFACl‐treated film (38.7%), indicating that the APFACl treatment effectively retards Sn^2+^ oxidation.

Moreover, the APFA adsorption not only passivates halide and Sn‐related defects, but also reinforces the lattice structure by increasing the formation energy of anti‐site defects including Pb_I_/Sn_I_ (Pb/Sn substituted in I position), and I_Pb_/I_Sn_ (I substituted in Pb/Sn position, Figure S13, Supporting Information). Furthermore, water contact angle measurements show that APFACl‐treated perovskite film significantly increases in contact angle from 65.8° to 84.8°, indicating the enhanced surface hydrophobicity (Figure S14, Supporting Information) by 1D perovskitoid, which effectively protects the perovskite film from the adsorption and infiltration of moisture molecules. Collectively, the surface‐oriented 1D perovskitoids can improve the light and humidity stability of Sn‐Pb perovskite films.

**Figure 4:**
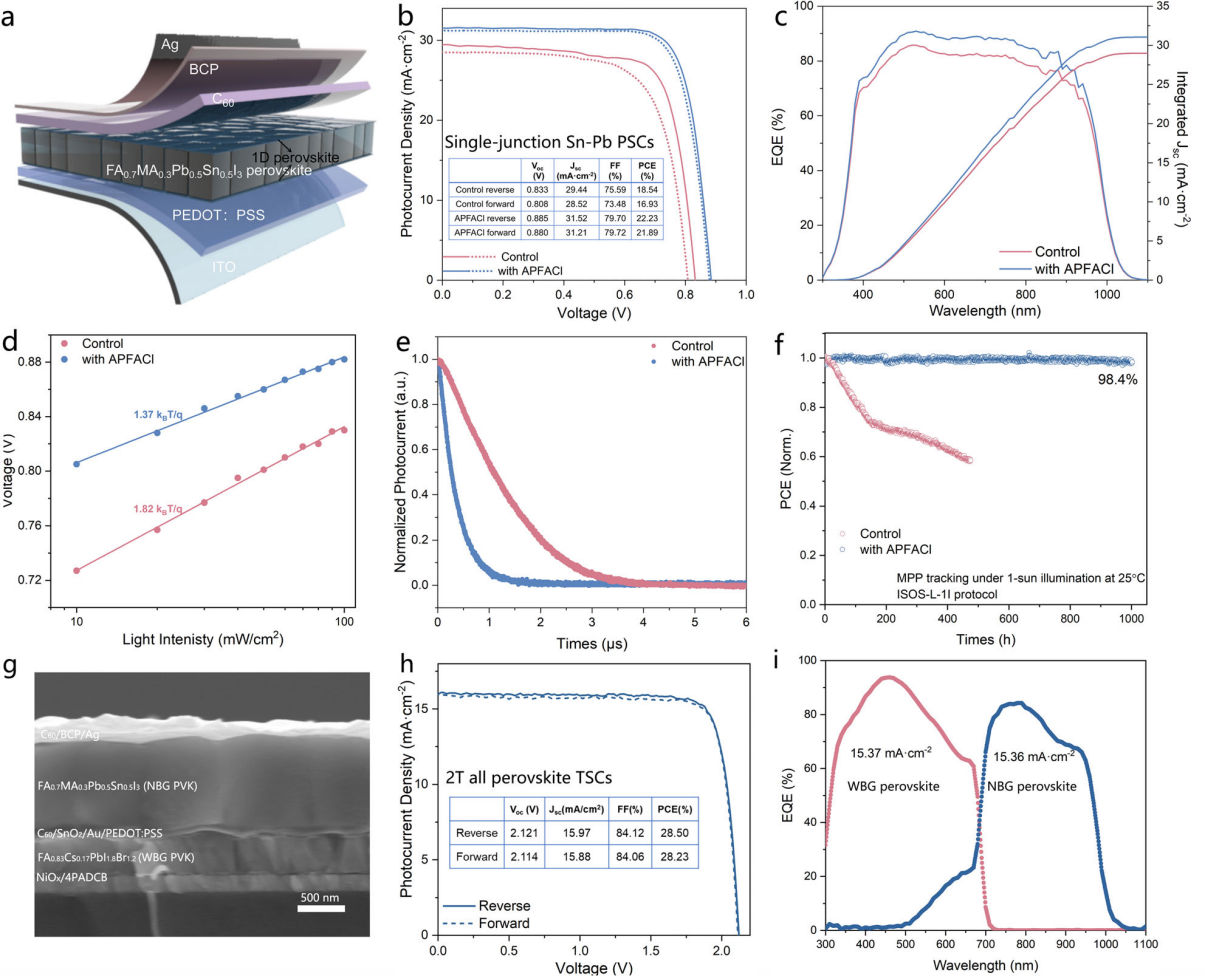
Sn‐Pb single‐junction and all‐perovskite tandem devices. a) Schematic diagram of devices based on 1D/3D heterostructure. b) *J‐*‐*V* curves of Sn‐Pb single‐junction devices under forward and reverse scan direction. c) EQE spectra of Sn‐Pb single‐junction devices. d) *V*_oc_ versus light‐intensity plots. e) Transient photocurrent decay curves of control and APFACl‐treated devices. f) MPP tracking of encapsulated Sn‐Pb single‐junction device under 1‐sun illumination (white LED) in N_2_ at 25 °C. g) Cross‐sectional SEM image of all‐perovskite TSCs (The composition of WBG perovskite (1.77 eV) and NBG perovskite (1.26 eV) are FA_0.83_Cs_0.17_PbI_1.8_Br_1.2_ and FA_0.7_MA_0.3_Pb_0.5_Sn_0.5_I_3_, respectively.) h) *J‐*‐*V* curves of all‐perovskite TSCs based on 1D/3D Sn‐Pb heterostructure under forward and reverse scan direction. i) EQE spectra of all‐perovskite TSCs.

To clarify the effect of spontaneously assembled dual‐interface 1D/3D Sn‐Pb perovskite heterostructure on the device performance, single‐junction Sn‐Pb based PSCs based on configuration of ITO/PEDOT: PSS/ FA_0.7_MA_0.3_Pb_0.5_Sn_0.5_I_3_/C_60_/BCP/Ag were fabricated (the device architecture is shown in **Figure** **4**a). Current density–voltage (*J‐* *V*) curves under forward and reverse scanning directions are presented in Figure 4b. The control device shows a power conversion efficiency (PCE) of 18.54% while the APFACl‐treated devices exhibit a significantly enhanced PCE of 22.23% (*V*_oc_ of 0.885 V, *J*_sc_ of 31.52 mA·cm^−2^, and FF of 79.72%). Figure 4c shows the external quantum efficiency (EQE) spectra of the control and APFACl‐treated device, with the integrated *J*_sc_ values of 28.99 and 31.05 mA·cm^−2^ respectively, in good agreement with the *J‐*‐*V* characteristics. Tracking under the applied biases of 0.68 V and 0.74 V, the control and APFACl‐treated devices present stabilized steady‐state power outputs (SPO) of 17.92% and 21.93%, respectively, demonstrating the reliable photovoltaic performance (Figure S15, Supporting Information). Statistical analysis shows that devices treated with 1 mol% APFACl achieve a significantly highest average PCE of 21.58% (Figure S16, Supporting Information), along with a narrower distribution, indicating excellent reproducibility.

Then we investigated the charge carrier recombination dynamics within the single‐junction Sn‐Pb based devices. As shown in Figure 4d, the linear relationship of *V*_oc_ of the devices with the natural logarithmic of light intensity ($V_{\mathrm{oc}}\propto \frac{nk_{B}\mathrm{Tln}(I)}{q}$) show that the ideality factor (n) decreases from 1.82 to 1.37, validating the markedly reduced trap‐assisted nonradiative recombination in APFACl‐treated devices.^[^ ^38^ ^]^ Furthermore, transient photocurrent/photovoltage measurements (TPC/TPV) were implemented to gain in‐depth insight into the charge extraction and recombination dynamics within the devices under operational conditions. The charge extraction time (*τ*_ext_) and recombination lifetime (*τ*_rec_) of photogenerated charge carriers within devices are obtained by fitting the TPC/TPV curves with mono‐exponent function.^[^ ^39^, ^40^, ^41^ ^]^ TPC measurements in Figure 4e show that the charge transport lifetime (*τ*_ext_) is reduced from 1.18 µs for control device to 0.347 µs for the APFACl‐treated device, corroborating the facilitated charge extraction from the perovskite layer to the charge transport layers within the devices. Meanwhile, the charge recombination lifetime (*τ*_rec_) extracted from TPV measurements increases from 390 µs to 476 µs, indicating the suppressed charge recombination and reduced trap density within the devices (Figure S17, Supporting Information). Collectively, the dual‐interface 1D/3D Sn‐Pb heterostructures effectively ameliorate the defect‐assisted recombination and facilitate the charge extraction from the perovskite absorber to the transport layers.

Figure 4f shows the long‐term operational stability of Sn‐Pb PSCs under maximum power point (MPP) tracking, conducted under 1‐sun illumination (white LED, 25 °C), in accordance with the ISOS‐L‐1I protocol. The control device exhibited pronounced degradation, with its PCE declining to approximately 60% of the initial value within 400 hours. In sharp contrast, the 1D/3D device demonstrated exceptional stability, retaining 98.4% of its initial efficiency even after 1000 hours of continuous operation. This outstanding result positions the APFACl‐treated device among one of the most stable Sn‐Pb PSCs reported to date (see Table S3 Supporting Information). The significant enhancement in operational stability is attributed to the formation of structurally stable 1D/3D perovskite heterostructure at both fragile interfaces. The contiguous 1D perovskitoid interphases encapsulating the 3D bulk reinforce the structural integrity of the Sn‐I octahedral frameworks, effectively mitigating I^−^ ion migration and Sn^2+^ oxidation, which are the main issues exacerbating the instability of Sn‐Pb based PSCs.

By integrating the optimized NBG PSCs with the 1.77 eV wide‐bandgap (WBG, FA_0.8_Cs_0.2_PbI_1.8_Br_1.2_) top subcells, we fabricated two‐terminal (2T) all‐perovskite TSCs with the architecture of ITO/NiO_x_/4PADCB/ FA_0.8_Cs_0.2_PbI_1.8_Br_1.2_/C_60_/ALD‐SnO_x_/Au/PEDOT: PSS/FA_0.7_MA_0.3_Pb_0.5_Sn_0.5_I_3_/C_60_/BCP/Au, as illustrated in Figure 4g. The all‐perovskite TSCs with 1D/3D Sn‐Pb PSCs delivered a remarkable PCE of 28.50%, accompanied by a *V*_oc_ of 2.121 V, a *J*_sc_ of 15.97 mA·cm^−2^, and an FF of 84.12% (Figure 4h). The integrated *J*_sc_ values obtained from EQE spectra for the WBG and NBG subcells were 15.37 and 15.36 mA·cm^−2^, respectively (Figure 4i). We continuously monitored the operational stability of the fabricated all‐perovskite TSCs based on 1D/3D Sn‐Pb heterostructure. The operational stability of the encapsulated tandem devices was evaluated under MPP tracking (N_2_, 25 °C) (Figure S18 Supporting Information). The encapsulated all‐perovskite TSC with 1D/3D Sn‐Pb heterostructure retained 90.37% of its initial PCE for approximately 600 hours, highlighting its excellent long‐term stability. These results demonstrate the critical role of dual‐interface 1D/3D perovskite heterostructure in improving the structure stability and mitigating performance losses of single‐junction Sn‐Pb PSCs and all‐perovskite TSCs.

## 3 Conclusion

In summary, 4‐aminobenzamidine dihydrochloride was introduced to spontaneously construct the dual‐interface 1D/3D perovskite heterostructure. Intermolecular π‐π stacking 1D perovskitoid serves as the nucleation site to modulate the perovskite crystallization, thus alleviating both inter‐/intra‐aggregated grain boundaries and tensile strains. Meanwhile, the dual‐interface orientally aggregated 1D perovskitoid serves as the full‐scale passivation and mechanically protective layer of 3D Sn‐Pb perovskite. The 1D/3D Sn‐Pb perovskite‐based devices achieved a high PCE of 22.23% along with a T_98_ operational lifetime over 1000 hours. Furthermore, all‐perovskite TSCs with the structurally reinforced 1D/3D Sn‐Pb heterojunction delivered a remarkable PCE of 28.50%, accompanied by a T_90_ operational lifetime of 600 hours. This work unlocks the full potential of mix‐dimensional perovskite heterostructure for enhancing the operational stability of Sn‐Pb PSCs and all‐perovskite tandem photovoltaics.

## Conflict of Interest

The authors declare no conflict of interest.

## Supporting information



Supporting Information

## Data Availability

The data that support the findings of this study are available from the corresponding author upon reasonable request.
