# Supplementary material for: Thermodynamically Reinforced Dual‐Interface 1D/3D Tin‐Lead Perovskite Heterostructure for Stable All‐Perovskite Tandem Solar Cells
Source: Adv Sci (Weinh). 2025 Dec 30;13(7):e17732. doi: 10.1002/advs.202517732 (PMC12866727; doi:10.1002/advs.202517732)
Supplement: Supplementary file 1 — Supporting Information [file ADVS-13-e17732-s001.docx]

Supporting Information for

**Thermodynamically Reinforced Dual-interface 1D/3D Tin-Lead Perovskite Heterostructure for Stable All-perovskite Tandem Solar Cells**

Hui Li ^a^, Zhen Liu ^a^*, Bohong Chang^a^, Yutong Wu^a^, Ruiyang Yin^b^*

^a^Key Laboratory for Liquid-Solid Structural Evolution and Processing of Materials, Ministry of Education, School of Materials Science and Engineering, Shandong University, Jinan 250061, P. R. China

^b^School of Materials Science and Engineering, Peking University, Beijing, China.

*To whom correspondence should be addressed. E-mail: yinry0228@163.com; [liuzhen7072@163.com](mailto:liuzhen7072@163.com)

**Contents**

1. **Experimental Section**
2. **Supporting Tables: Table S1 to S3**
3. **Supporting Figures: Figure S1 to S18**
   - - 1. **Experimental Section**
   1. **Perovskite Precursor Solution preparation.** **NBG perovskite:** 1.8 M FA_0.7_MA_0.3_Pb_0.5_Sn_0.5_I_3_ precursor solution was prepared by mixing 1.26 M FAI, 0.54 M MAI, 0.9 M PbI_2_, 0.9 M SnI_2_, 0.045 M SnF_2_, 1.8 mg/mL GlyHCl (Glycine hydrochloride) and 3 mg/mL Pb(SCN)_2_ in co-solvent DMSO/DMF (1 mL, 1:3 volume ratio). Then 5 mg tin powder was added into the precursor solution and vigorously stirred for 4 h to reduce Sn^4+^. Finally, the precursor solution was filtered by 0.22 µm PTFE filter. **WBG perovskite:** 1.2 M FA_0.83_Cs_0.17_Pb(I_0.6_Br_0.4_)_3_ precursor solution was prepared by mixing FAI, CsI, PbI_2_, PbBr_2_, PbSCN_2_ with the molar ration of 0.83:0.17:0.40:0.60:0.01 in co-solvent DMSO/DMF (1 mL, 1:3 volume ratio). The perovskite precusor solutions were stirred at the room temperature for overnight and filtered by 0.22 µm PTFE filter before using.

**1.2 Synthesis of 1D APFAPbI_4_ single crystal.** PbO (111.57 mg) and SnO powder (67.35 mg) were dissolved in a mixed solvent of H_3_PO_2_ aqueous solution (1.7 mL) and aqueous HI solution (3.4 mL) by heating and stirring until the solution turned clear yellow. After cooling to room temperature, APFACl (1 mmol, 208.09 mg) was directly added to the above solution, and heating and stirring until the solid gradually dissolved to form clear yellow solution. Then yellow needle-like crystals precipitated during the solution slow cooling process. Under N_2_ atmosphere, 1D APFAPbI_4_ single crystal were isolated by suction filtration and dried sufficiently on a hot plate at 70°C.

**1.3 Device fabrication.** The patterned glass/ITO substrates were cleaned by detergent, deionized water, acetone, and isopropanol in 15 minutes ultrasonic bath respectively, and then dried with N_2_ flow, followed by UV-Ozone treatment. PEDOT: PSS layer was fabricated by an aqueous dispersion (filtered by a 0.45 µm PVDF filter) and then spin-coated on the glass/ITO substrate at 4700 rpm for 30s, followed by annealing in air at 150°C for 20 min. Then transferring to N_2_ glovebox and degassing at 150°C for 30 min. The perovskite layer was deposited by spin-coating above precursor solution with two-step spin-coating procedures: 1000 rpm for 10s and 4000 rpm for 40 s. Chlorobenzene (400 µl) as antisolvent was rapidly dripped onto the spinning substrate over an interval of 1 s during the second spin-coating step at 10 s before the end of the procedure, then annealing at 100°C for 10 min. EDAI_2_ (1 mg/mL in isoproposal) was sequentially spin-coated at 5,000 rpm for 30 s. The fabricated substrates were subsequently transferred to a vacuum chamber where 25 nm C_60_ (deposition rate of 0.01 nm·s^-1^), 5 nm BCP (deposition rate of 0.01 nm·s^-1^) and 120 nm Ag electrode were consecutively deposited by thermal evaporation on the above substrate to the perovskite solar cells.

The architecture of the all-perovskite tandem solar cells employed a monolithic two-terminal configuration with the following layer sequence: ITO/NiO_x_/4PADCB/WBG perovskite/C_60_/SnO_2_/Au/PEDOT: PSS/LBG perovskite/C_60_/BCP/Ag. The wide-bandgap (WBG) perovskite top subcell was fabricated following: A NiO_x_ nanocrystal solution (10 mg/mL in deionized water) was spin-coated onto ITO substrates at 3000 rpm for 40 s, followed by thermal annealing at 110 °C for 15 min on a hotplate in ambient air. After thermal annealing, the substrates were promptly transferred into a N_2_-filled glovebox. Subsequently, 4PADCB solution (0.4 mg/mL) was drop-cast onto the spinning ITO/NiO_x_ substrates at 4000 rpm for 30 s. The resulting self-assembled monolayer was thermally annealed at 100 °C for 10 min. Then Al_2_O_3_/isoproposal was dispensed onto the annealed films while dynamically spin-coating at 4,000 rpm for 30 s. The substrates were then dried on a 100°C hotplate for 20 min. For post-treatment, EDAI₂ (1 mg/mL in IPA) was carried out sequentially via spin-coating at 5,000 rpm for 30 s. After each post-treatment, the films were annealed at 100 °C for 5 min. Upon cooling to room temperature, the substrates were transferred to a thermal evaporation chamber. A 20 nm C_60_ electron transport layer was then deposited at a rate of 0.01 nm·s^-1^ under high vacuum. The interconnecting layer, serving as both an optical spacer and recombination layer, consisted of a 20 nm SnO_2_ layer deposited via atomic layer deposition (ALD) at 70 °C, followed by a 1 nm Au layer formed by thermal evaporation. The fabrication of the NBG perovskite bottom subcell was performed using the above procedure. Finally, all devices were encapsulated with a UV-curable epoxy and cover glass to ensure long-term operational stability. The aperture area defined by the metal mask during *J*-*V* measurement is 0.0625 cm^2^. The total device area, defined by the overlapping area of the bottom and top electrodes, is approximately 0.09 cm^2^.

**1.4 Characterization**. Rigaku Smartlab 9kW was employed to conduct the X-ray diffraction (XRD) and grazing incidence XRD (GIXRD). Single crystal XRD data was collected by an Agilent Gemini/Xcalibur single-crystal diffractometer equipped with a graphite-monochromated Mo Kα radiation source (λ = 0.71073 Å) at 293 K. Field emission scanning electron microscopy (FESEM, SU-70) record the top-view and cross-sectional morphology images of perovskite films. Fourier transform infrared spectroscopy (FTIR, NEXUS670), ^1^H liquid-state nuclear magnetic resonance (NMR, Bruker 400 MHz) and X-ray photoelectron spectroscopy (Escalab 250Xi electron spectrometer) were conducted to elucidate the molecular interaction. PTIR measurements were measured by a NanoIR2-FS setup (testing range is from 900 to 1800 cm^-1^) consisting of an AFM microscope operating in contact mode. The surface potential and conduct current were characterized by SPM-9700 atomic force microscope in Potential Channel equipped with PPP-EFM probe. PFQNM-AFM were measured by Bruker BioScope Resolve atomic force microscope. Time-of-flight secondary-ion mass spectrometry (TOF-SIMS) measurements were conducted by ION-TOF SIMS 5. Specord Plus 200 Spectro-photometer record the UV-vis absorption spectra. Contact angle measurements were carried out by SDC-200S. Steady-state (PL) were characterized by FLS920 all functional fluorescence spectrometer (Edinburgh). SCLC measurements were performed by Keithley 2420 source measurement unit under dark. Current density-voltage (*J*-*V*) curves were measured by a Keithley 2450 analyzer under the stimulated AM 1.5 G illumination. Transient photovoltage/photocurrent (TPV/TPC) spectra were recorded by Tektronix MDO3032 with a wavenumber of 485 nm.

**1.5 DFT Calculation methods.** First-principles calculations utilize the Vienna Ab initio Simulation Package (VASP) based on density functional theory (DFT). The generalized gradient approximation (GGA) of Perdew-Burke-Ernzerhof (PBE) functional was utilized as the exchange-correlation functional. A plane-wave cutoff energy of 420 eV was utilized, and the vacuum region was set to 30 Å to avert the fictitious interaction with its periodic image. The energy and force convergence criteria were set to 10^-6^ eV and 0.05 eV Å^-1^, respectively. Defect formation energy was calculated based on s supercell with 3×3×3 periodicity and 1×1×1-center k-point mesh. All the calculations were based on the FA-I or Pb/Sn-I terminal on the thermodynamically stable (001) plane of FAPb_0.5_Sn_0.5_I_3_ crystal. The formation energy of a defect in charge state q is given by: *E*_f_ = *E*_defect_ - *E*_free_ -∑_𝑖_𝑛_𝑖_𝜇_𝑖_, where *E*_defect_ and *E*_free_ are the total energies of the defect and defect-free surfaces, respectively. *n*_i_ and 𝜇_𝑖_ are respective the number and chemical potential of added (*n*_i_>0) or removed (*n*_i_<0) for i-type atom. The adsorption energy of adsorbate molecules on the perovskite surface was calculated as: *E*_perovskite+molecule_ - *E*_perovskite_ - *E*_molecule_, where *E*_perovskite+molecule_, *E*_perovskite_, and *E*_molecule_ is the total energies of the absorption system, perovskite and molecules, respectively.

**1.6 Statistical analysis.** Detailed information is provided as follows: The photovoltaic parameters (including PCE, J_sc_, V_oc_, FF, and EQE) presented in Figure 4 are all raw data without any mathematical processing. The data presented in the manuscript primarily represent the most characteristic experimental results (e.g., the optimal J-V curve of the devices), aiming to clearly illustrate the observed phenomena and trends. Statistical methods used to assess significant differences with sufficient details: The data mainly derive from representative experiments showing key trends and phenomena. Group comparisons are based on direct numerical contrasts from single or few measurements. Therefore, all noted differences are descriptive and observational, not inferential.

**2. Supporting Tables**

**Table S1.** Crystal Data and Structure Refinement for APFABI_4_ (B = Sn, Pb).

| Empirical formula | C_7_H_12_N_3_BI_4_ (B = Sn, Pb) |
| --- | --- |
| Formulate weight | 853 |
| Space group | P2_1_/c |
| Unit cell dimensions | a=10.6129 Å |
|  | α = 90° |
|  | b = 19.2941 Å |
|  | β = 99.576 ° |
|  | c = 8.1854 Å |
|  | γ = 90° |
| Volume | 1652.74 Å^3^ |
| Z | 4 |
| Density | 3.428 g·cm^-3^ |
| h, k, l_max_ | 13, 24, 10 |

**Table S2.** TRPL fitting parameters of perovskite films.

|  | *τ*_1_ (ns) | *A*_1_ (%) | *τ*_2_ (ns) | *A*_2_ (%) | *τ*_avg_ (ns) |
| --- | --- | --- | --- | --- | --- |
| Control | 16.0 | 39.6 | 164.5 | 53.0 | 153.3 |
| With APFACl | 1955.5 | 35.6 | 9791.4 | 56.8 | 8895.0 |

**Table S3**. Comparison of PCE and operational stability of Sn-Pb PSCs.

|  | Bandgap  (eV) | PCE (%) | Operational stability  (ISOS-L-1I protocol) | Reference |
| --- | --- | --- | --- | --- |
| 1 | 1.23 | 22.31 | 86%~1000 hours | *ACS Energy Lett.***2023**, 8, 12, 5206-5214. |
| 2 | 1.25 | 22.7 | 80%~400 hours | *Adv. Energy Mater.* **2023**, 13, 2301218. |
| 3 | 1.23 | 23.7 | 88%~1000 hours | *Adv. Energy Mater.* **2023**, 13, 2204115. |
| 4 | 1.26 | 22.6 | 88%~1200 hours | *ACS Energy Lett.* **2023**, 2, 1068-1075. |
| 5 | 1.25 | 19.34 | 80%~358 hours | *Adv. Funct. Mater.***2024**, 34, 2306571. |
| 6 | 1.25 | 22.31 | 80%~800 hours | *Adv. Funct. Mater.* **2024**, 34, 2310828. |
| 7 | 1.26 | 23.44 | 88%~1000 hours | *Adv. Mater.* **2024**, 36, 2410298. |
| 8 | 1.25 | 23.32 | 82%~800 hours | *Angew. Chem. Int. Ed.***2024**, 63, e202409072. |
| 9 | 1.25 | 23.90 | 90%~650 hours | *Nat Energy***2024**, 9, 1388-1396. |
| 10 | 1.24 | 23.42 | 99.9%~420 hours | *Adv. Mater.***2024**, 36, 2405807. |
| 11 | 1.27 | 22.14 | 91%~550 hours | *Adv. Energy Mater.***2024**, 14, 2402171. |
| 12 | 1.25 | 23.34 | 75%~1000 hours | *Adv. Energy Mater.* **2024**, 14, 2304234. |
| 13 | 1.23 | 21.22 | 90%~1000 hours | *Angew. Chem. Int. Ed.***2024**, 63, e202317446*.* |
| 14 | 1.26 | 24.23 | 82%~450 hours | *Adv. Funct. Mater.* **2025**, 35, 2411746. |
| 15 | 1.37 | 17.4 | 90%~480 hours | *Adv. Mater.* **2025**, 37, 2414125. |
| 16 | 1.27 | 24.33 | 92%~340 hours | *Adv. Funct. Mater.* **2025**, 2421416. |
| 17 | 1.27 | 22.88 | 93%~500 hours | *Adv. Energy Mater.* **2025**, 15, 2403186. |
| 18 | 1.25 | 23.2 | 66%~140 hours | *Energy Environ. Sci.* **2025**,18, 439-453 |
| 19 | 1.25 | 23.76 | 89%~1100 hours | *Angew. Chem. Int. Ed.* **2025**, e202501188. |
| 20 | 1.26 | 22.7 | 81.35%~350 hours | *Adv. Mater.* **2025**, 37, 2415627 |
|  | 1.24 | 22.23 | **98.4%~1000 hours** | **This work** |

1. Supporting Figures


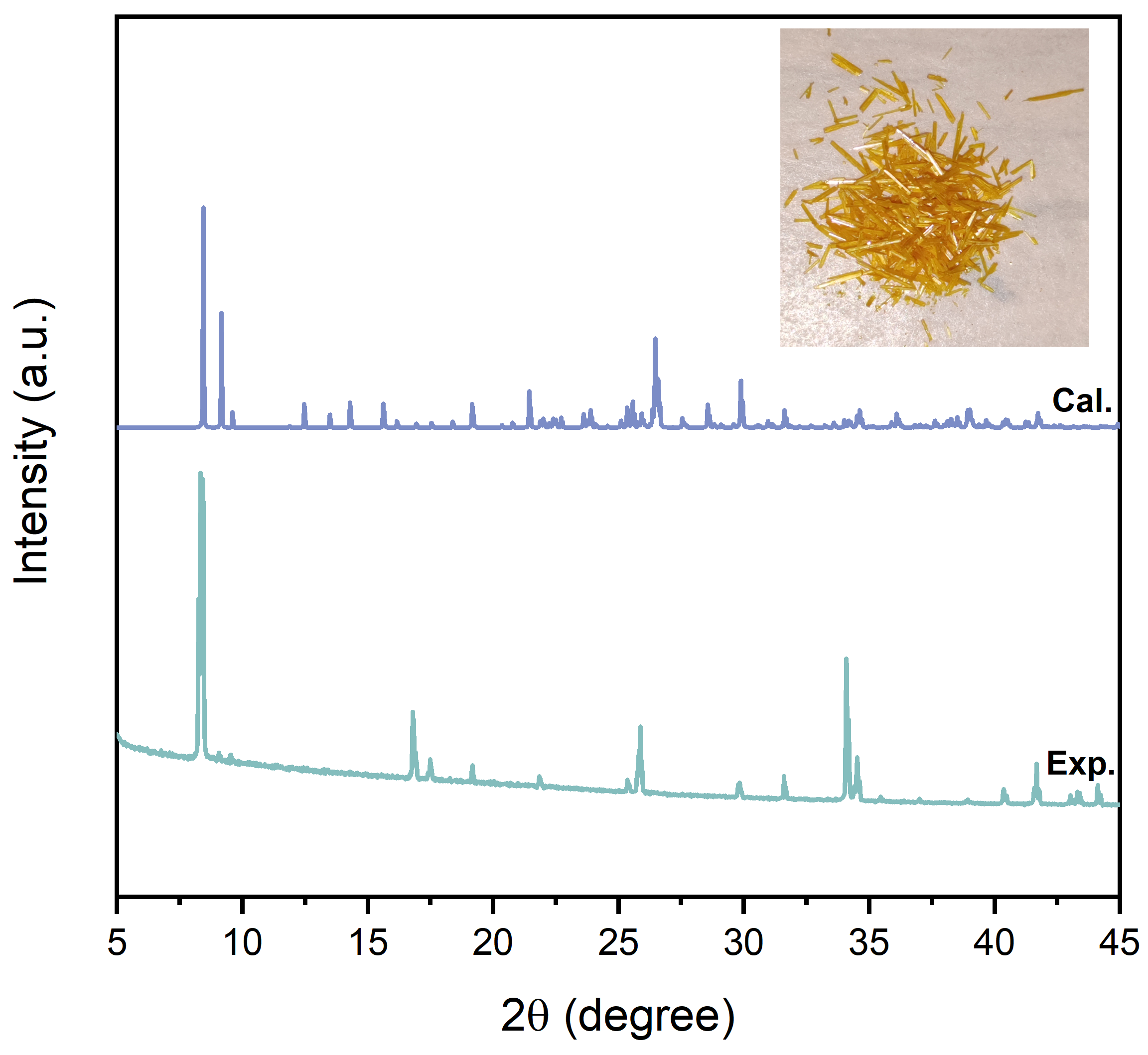


**Figure S1.** The calculated and experimental XRD patterns of 1D perovskitoid.





**Figure S2.** Tauc plot obtained from the UV-vis absorption spectra of 1D perovskite film.


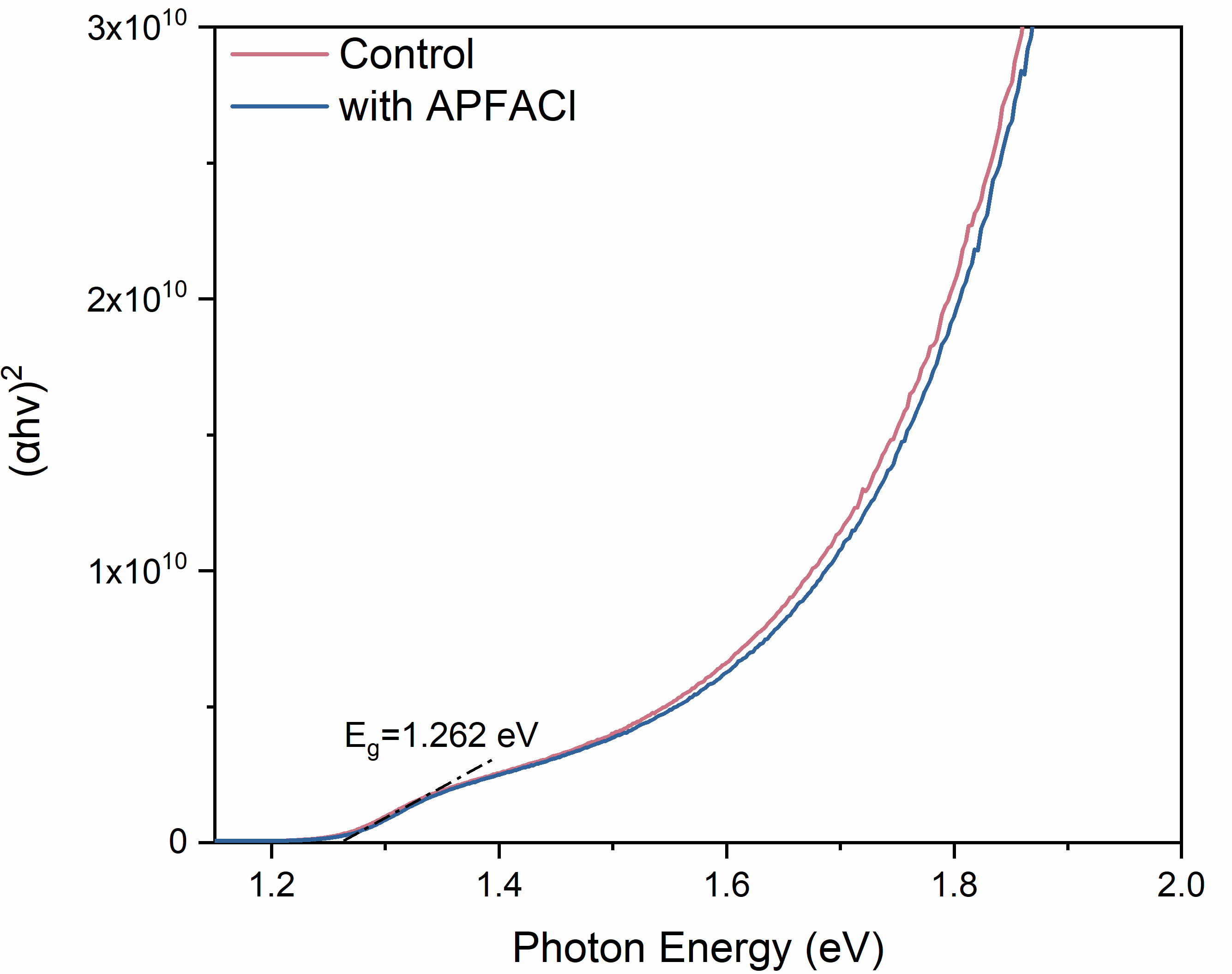


**Figure S3**. Tauc plot obtained from the UV-vis absorption spectra of Sn-Pb perovskite films without (control) and with APFACl addition.


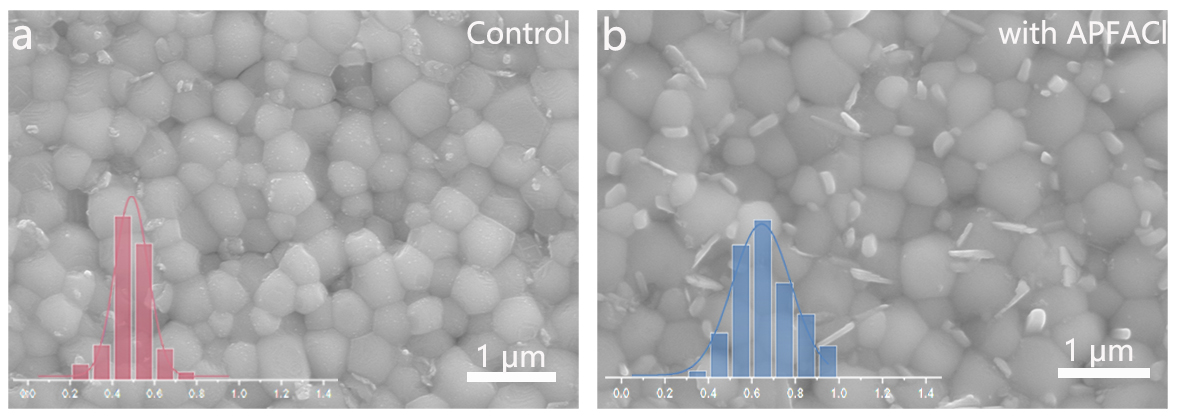


**Figure S4.** Top SEM images of perovskite films (a) without and (b) with 1 mol% APFACl addition. (The inset is the statistical analysis of grain size by Nano measurement.)


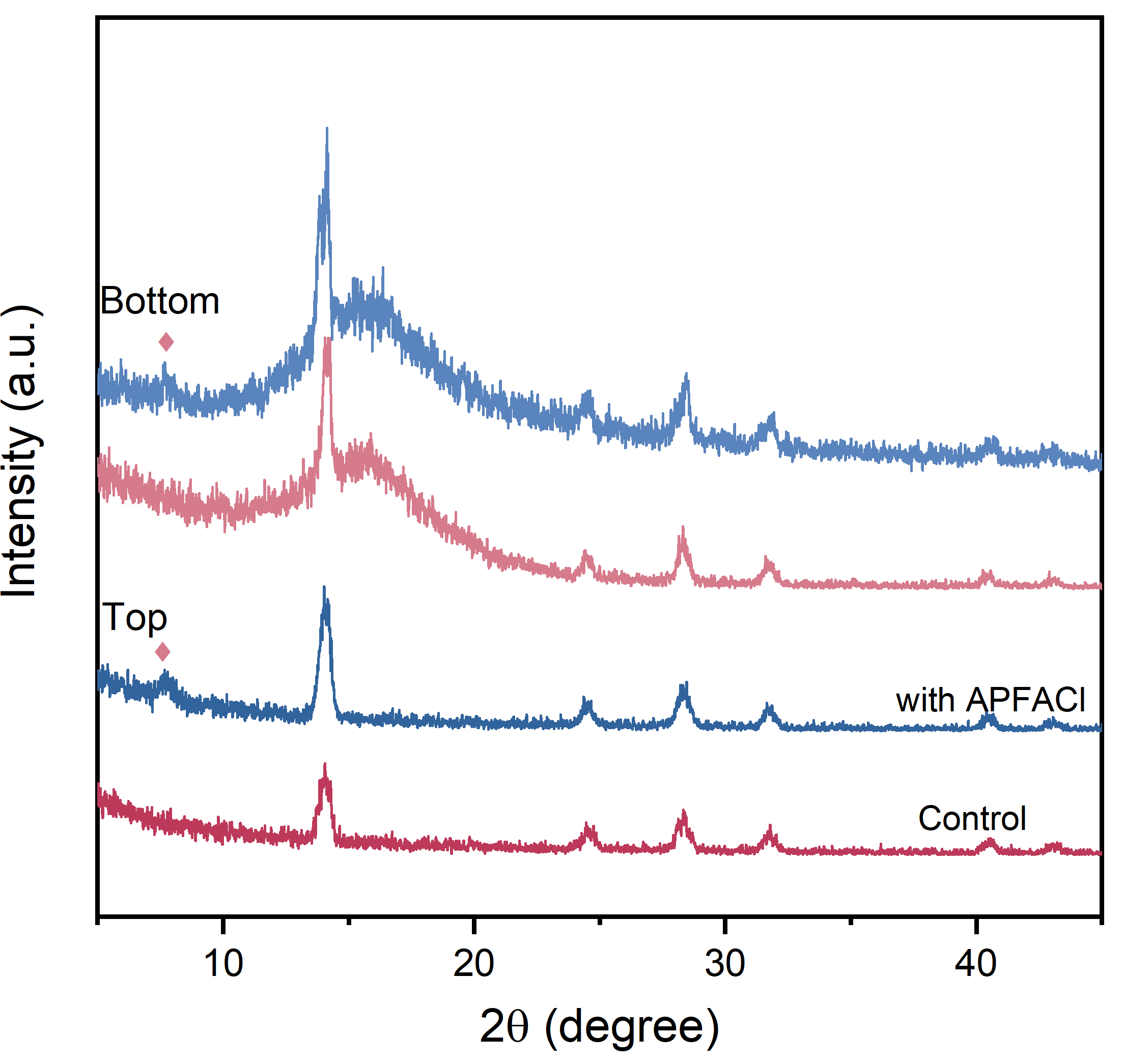


**Figure S5.** GIXRD patterns with 0.3° incident angle of perovskite film of top and bottom surface.


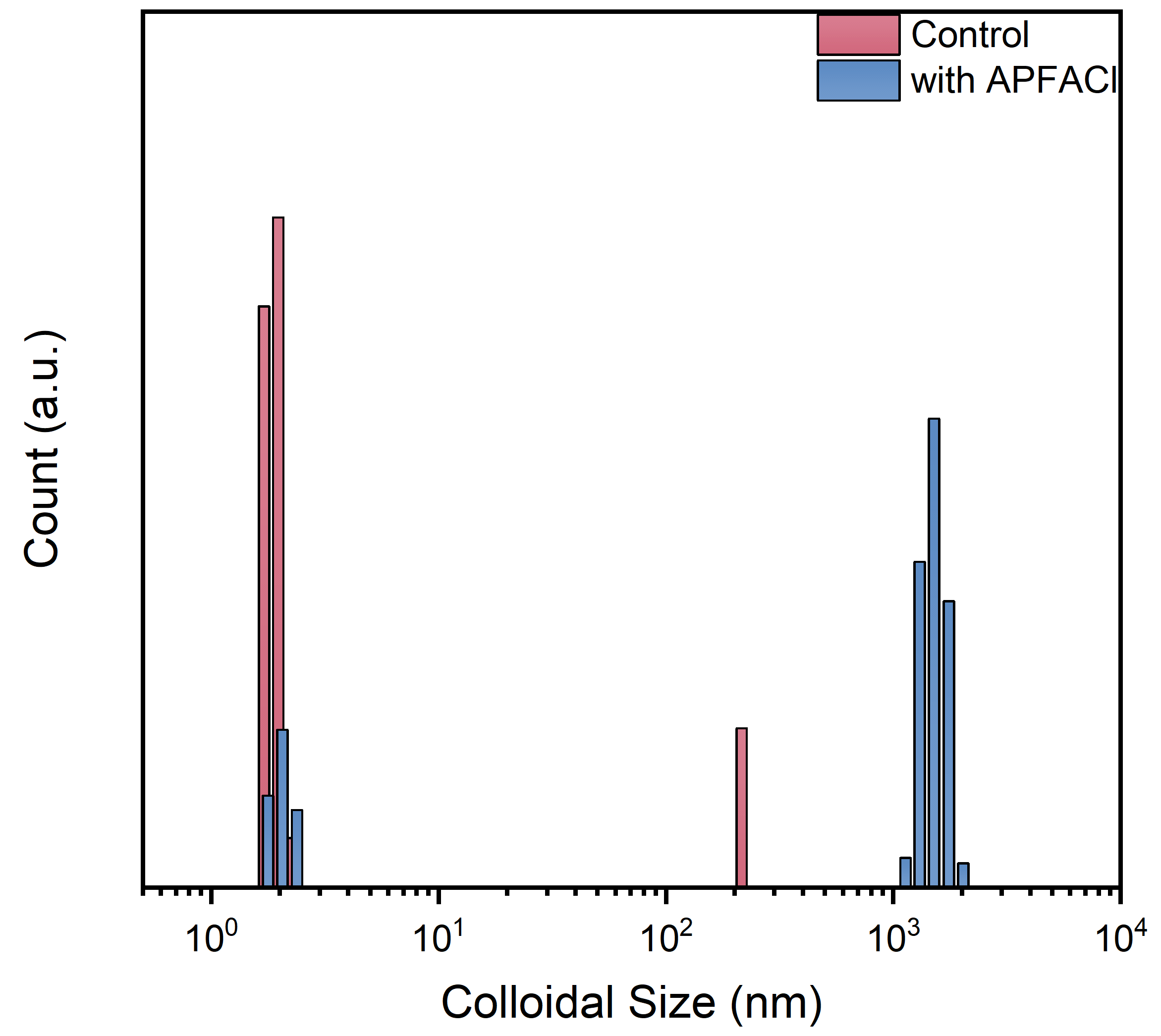


**Figure S6.** DLS spectra of perovskite precursor solution.


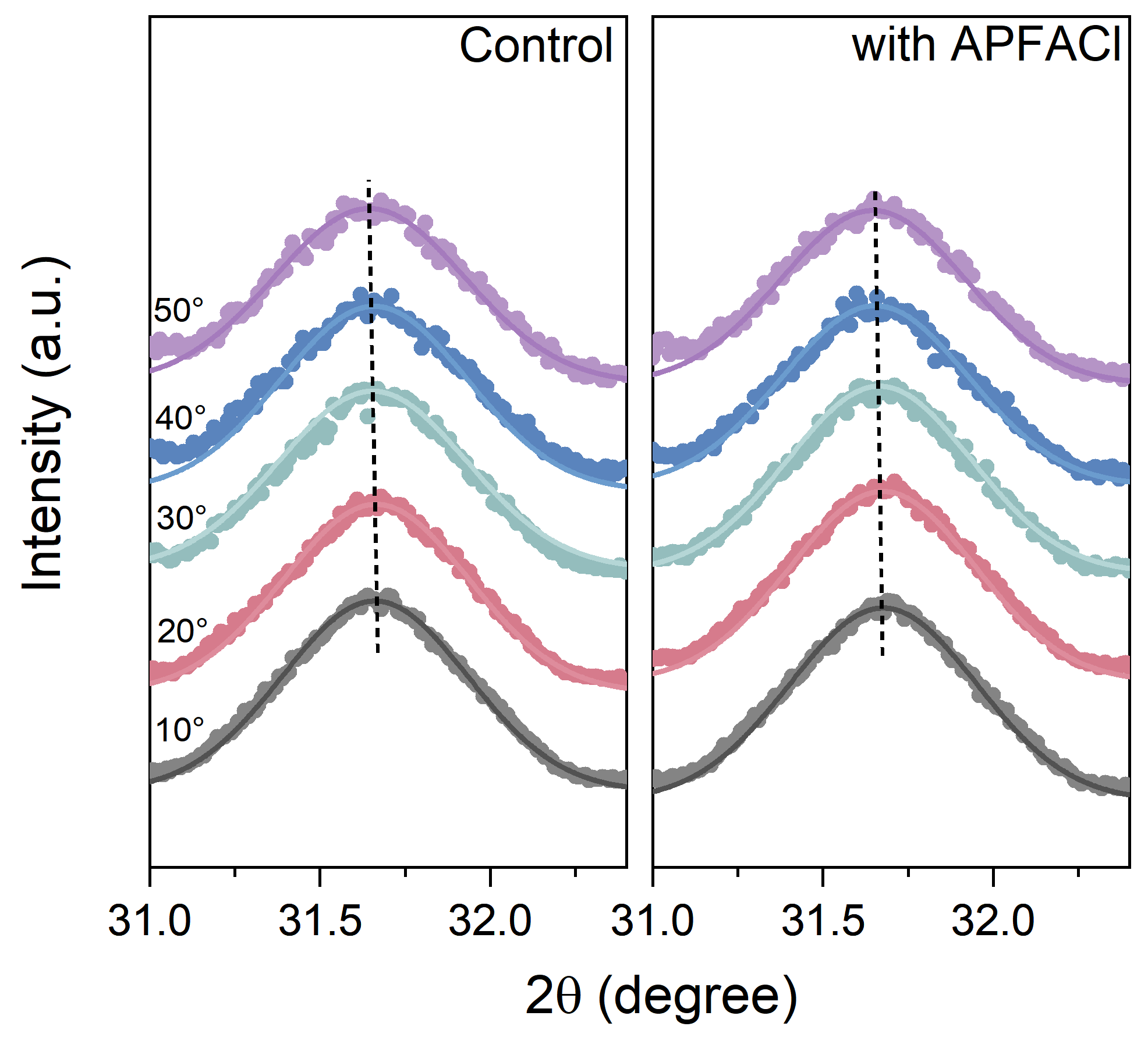


**Figure S7**. GIXRD patterns at different instrument tilt angles.


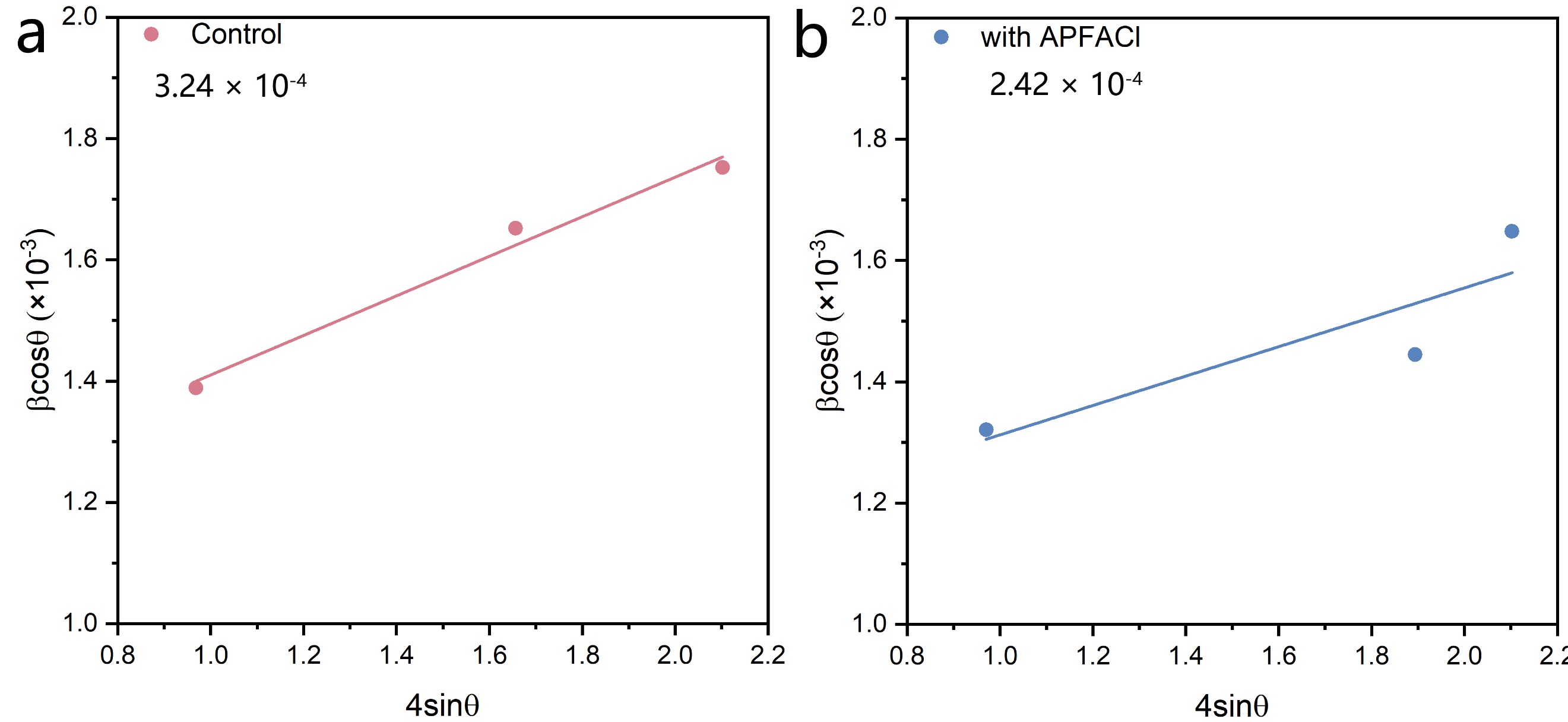


**Figure S8.** Williamson-Hall plot to determine the microstrain component of perovskite films fabricated (a) without and (b) with APFACl.


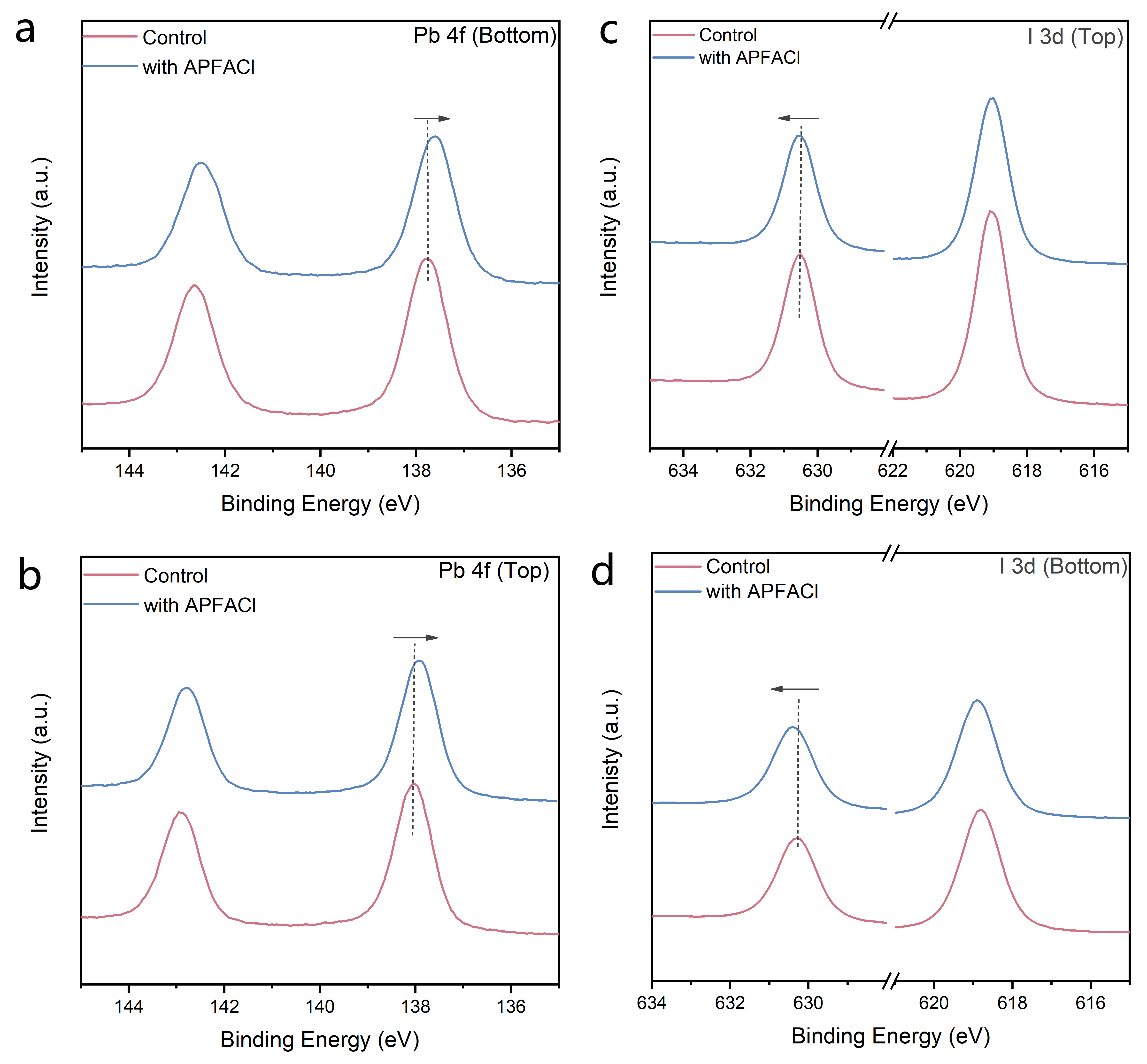


**Figure S9.** XPS spectra of (a, b) Pb 4f. (c, d) I 3d of top and bottom surface of perovskite films.


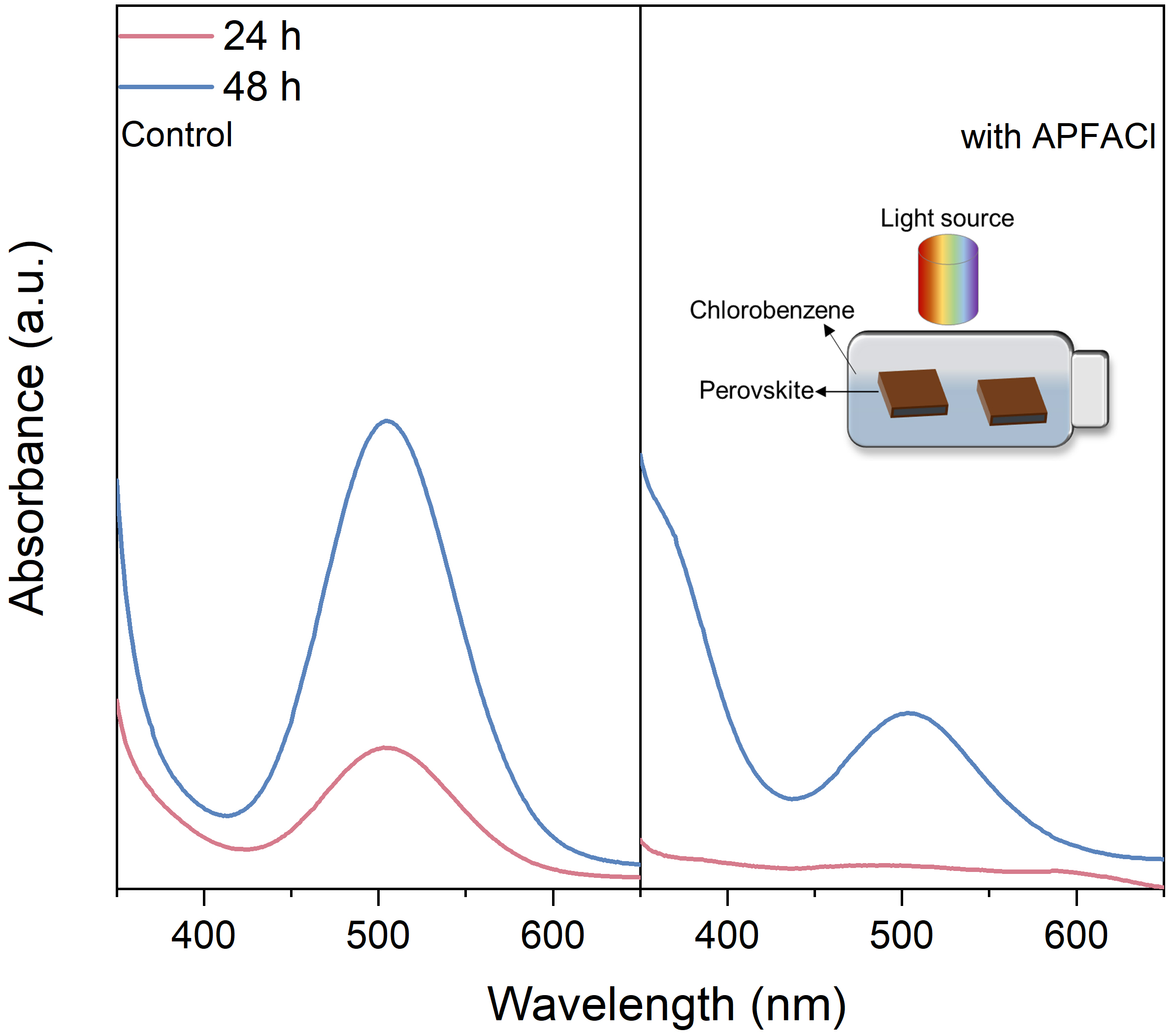


**Figure S10**. UV-vis absorbance spectra recorded for chlorobenzene solution taken from perovskite films aging under light exposure.


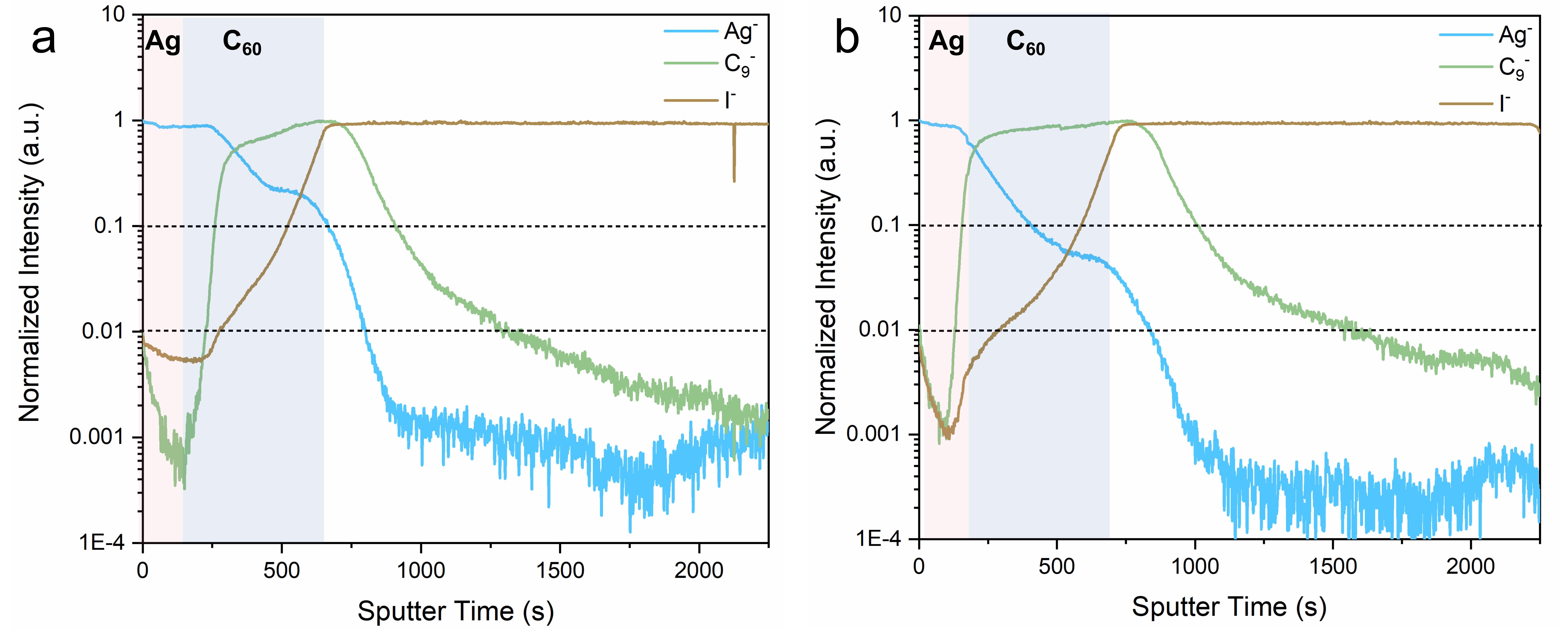


**Figure S11.** ToF-SIMs of aged devices (a) without (control) and (b) with APFACl addition.


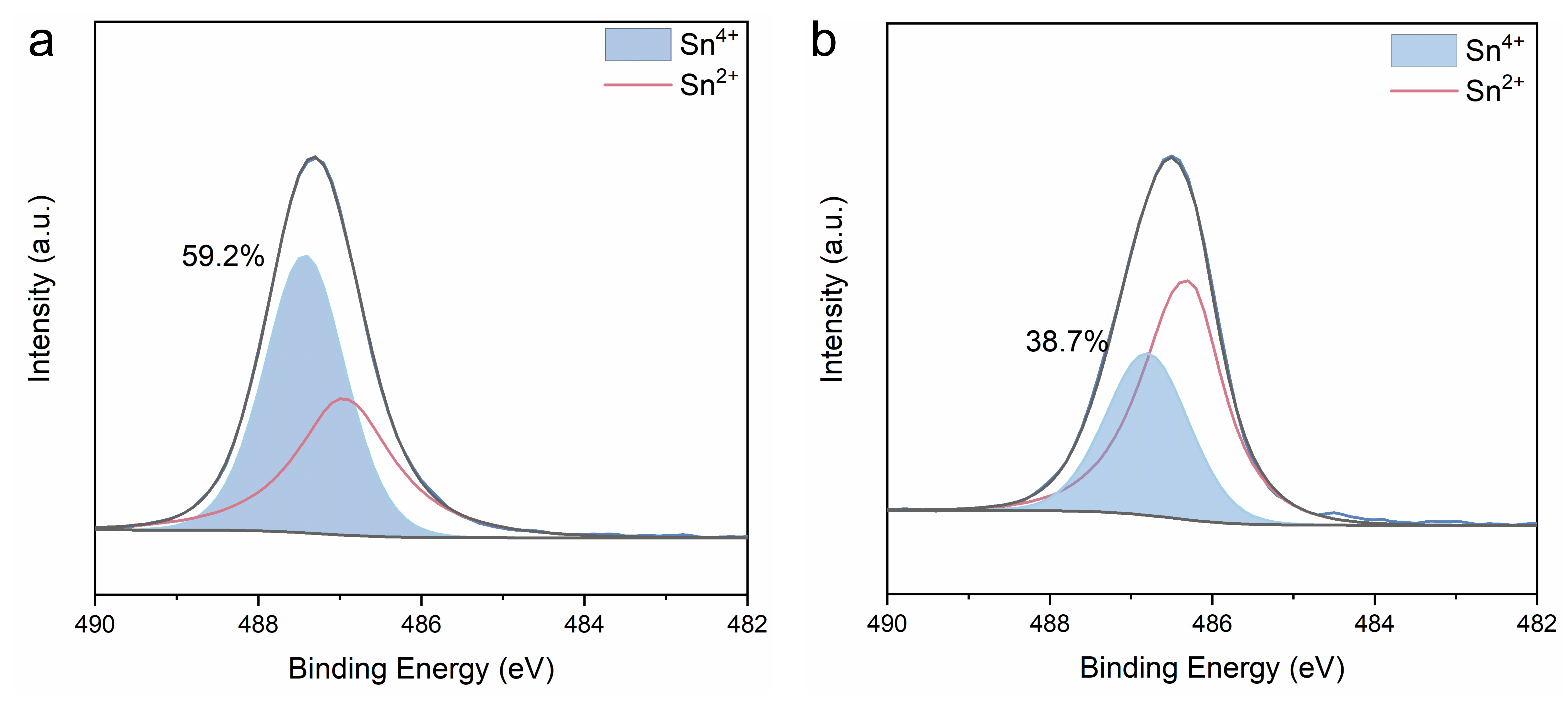


**Figure S12**. XPS spectra of Sn 4d of perovskite film (a) without and (b) with APFACl addition after oxidation in air.


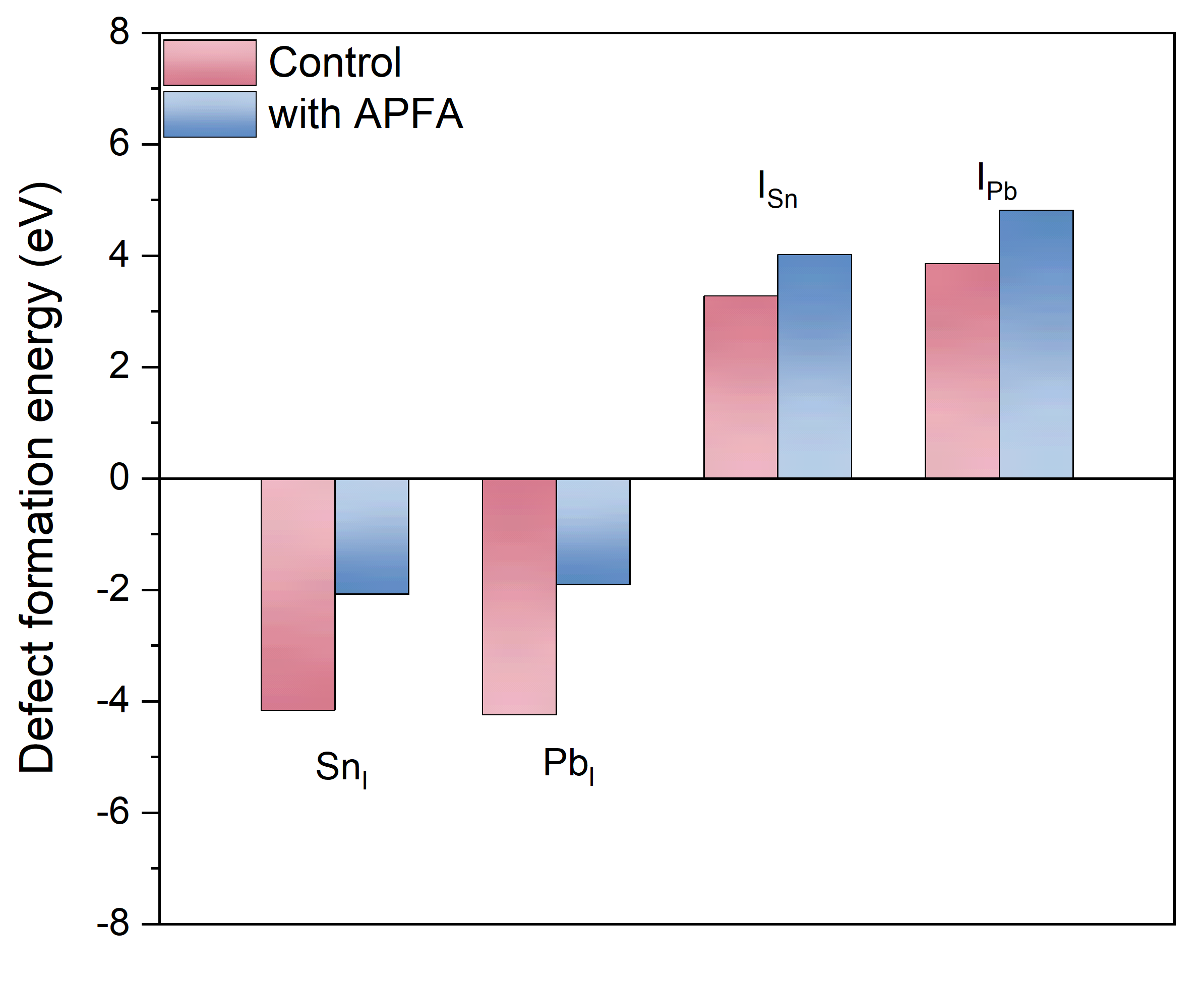


**Figure S13.** Defect formation energy of anti-site defects including Pb_I_/Sn_I_ and I_Pb_/I_Sn_


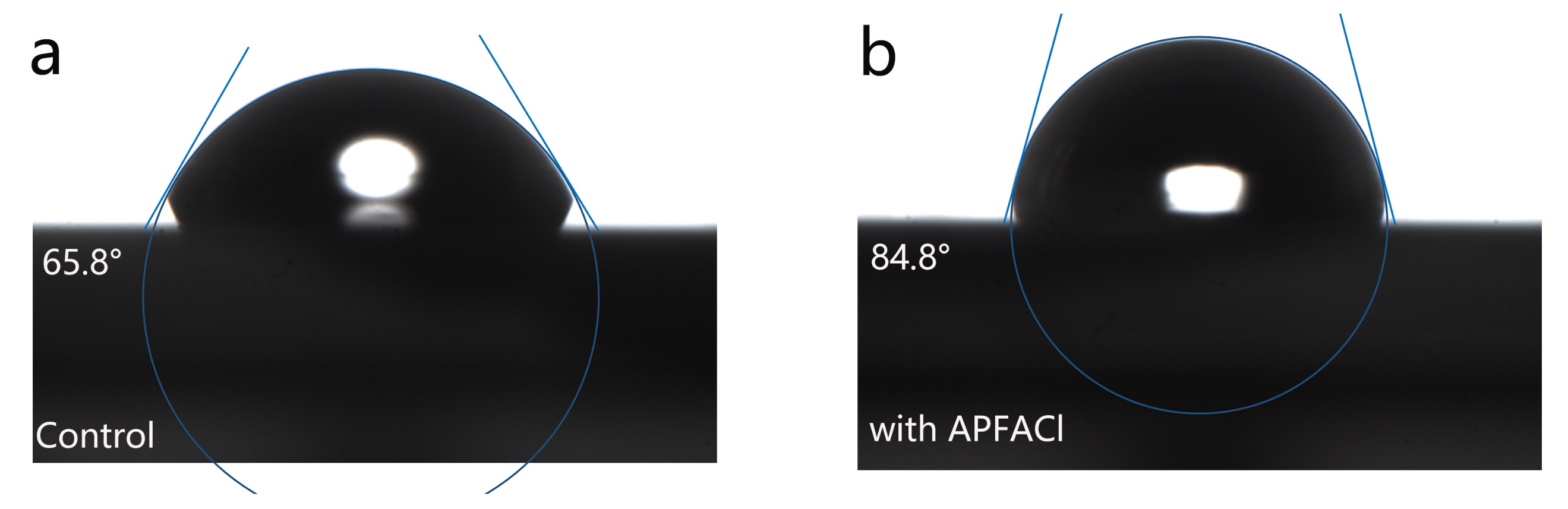


**Figure S14.** Water contact angle of perovskite film (a) without and (b) with APFACl addition.


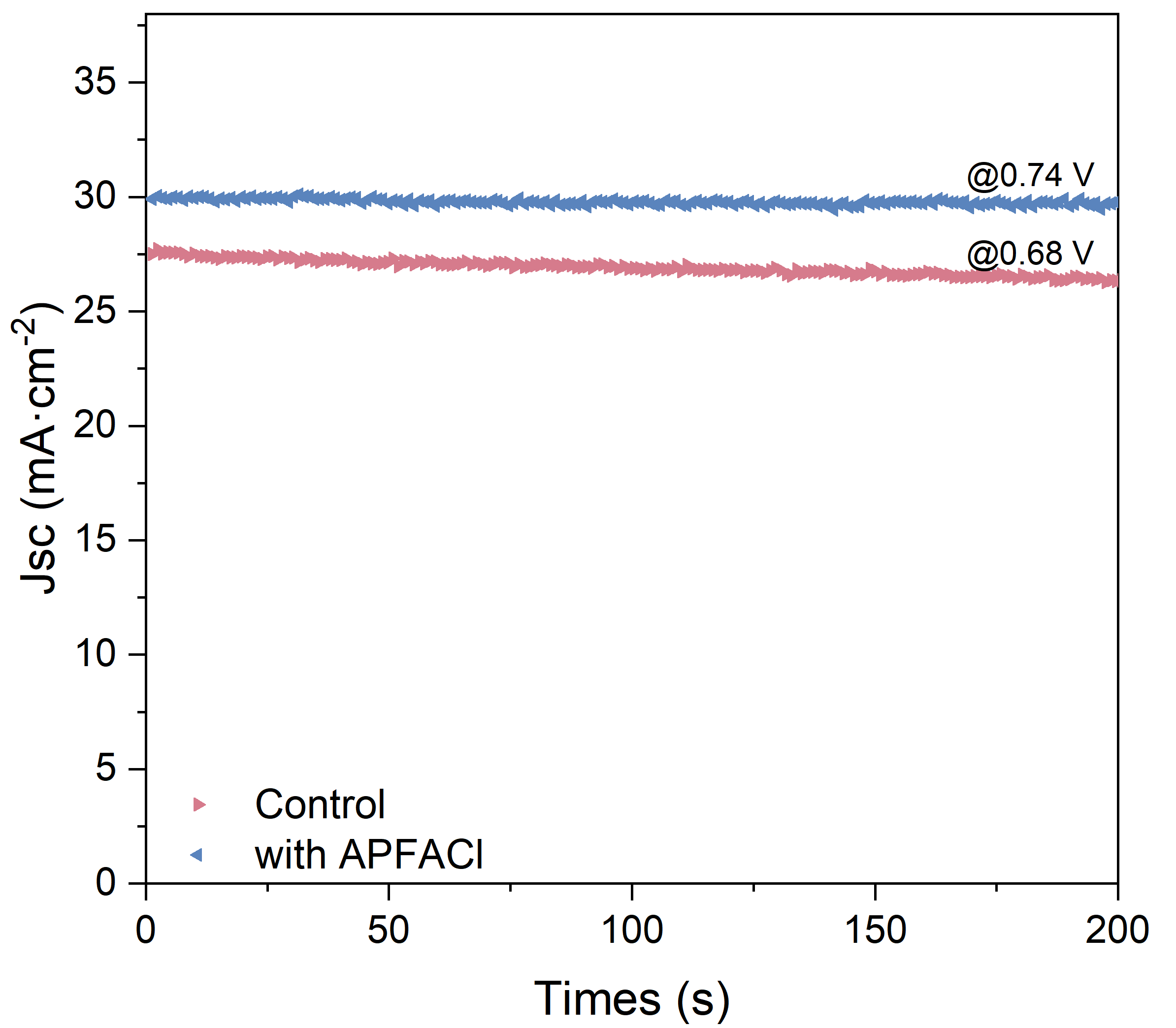


**Figure S15.** Steady-state photocurrent output of control and APFACl-treated devices.


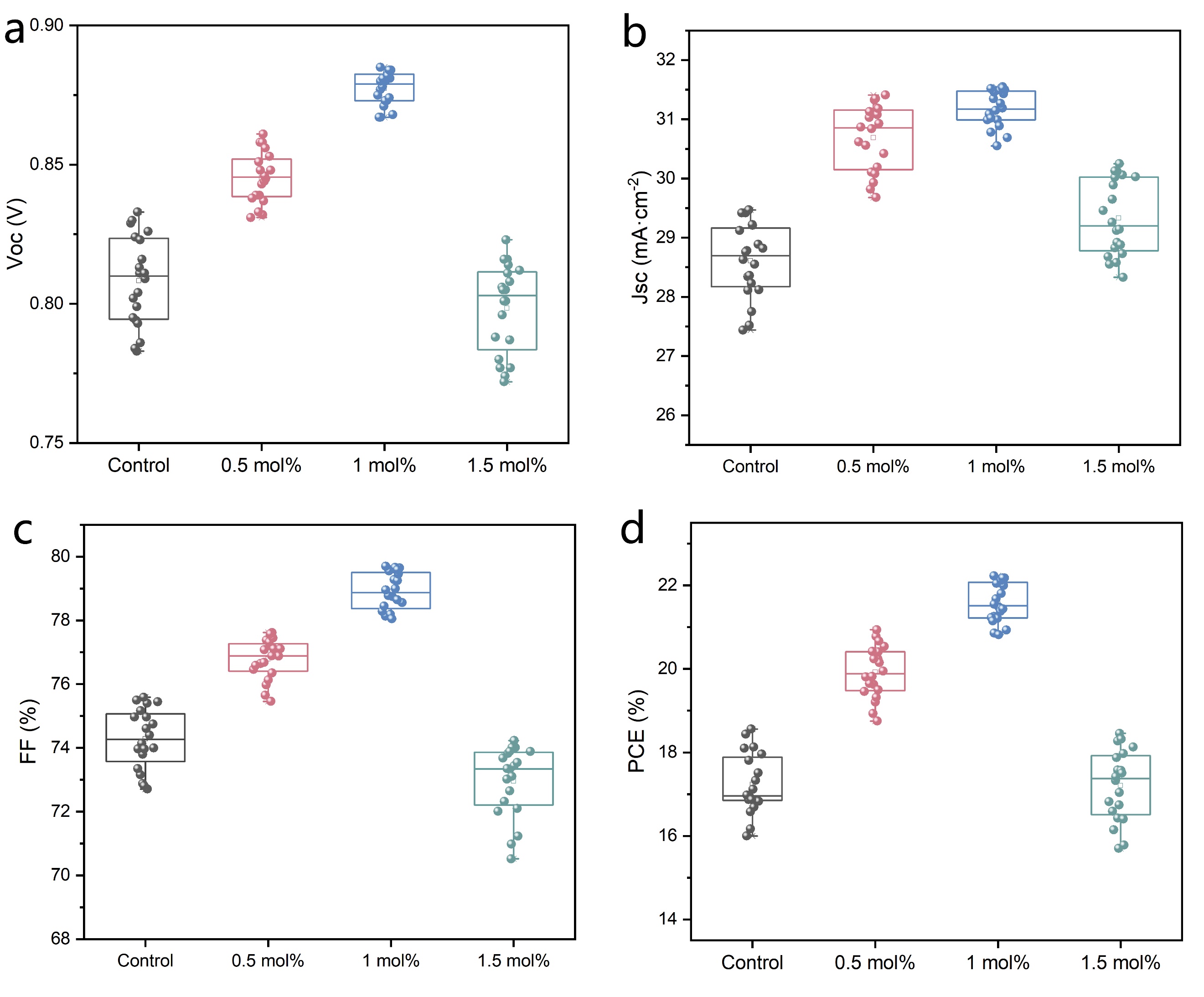


**Figure S16.** Photovoltaic performance parameters distribution (a) *V*_oc_, (b) *J*_sc_, (c) FF, and (d) PCE. Each condition includes 20 devices.


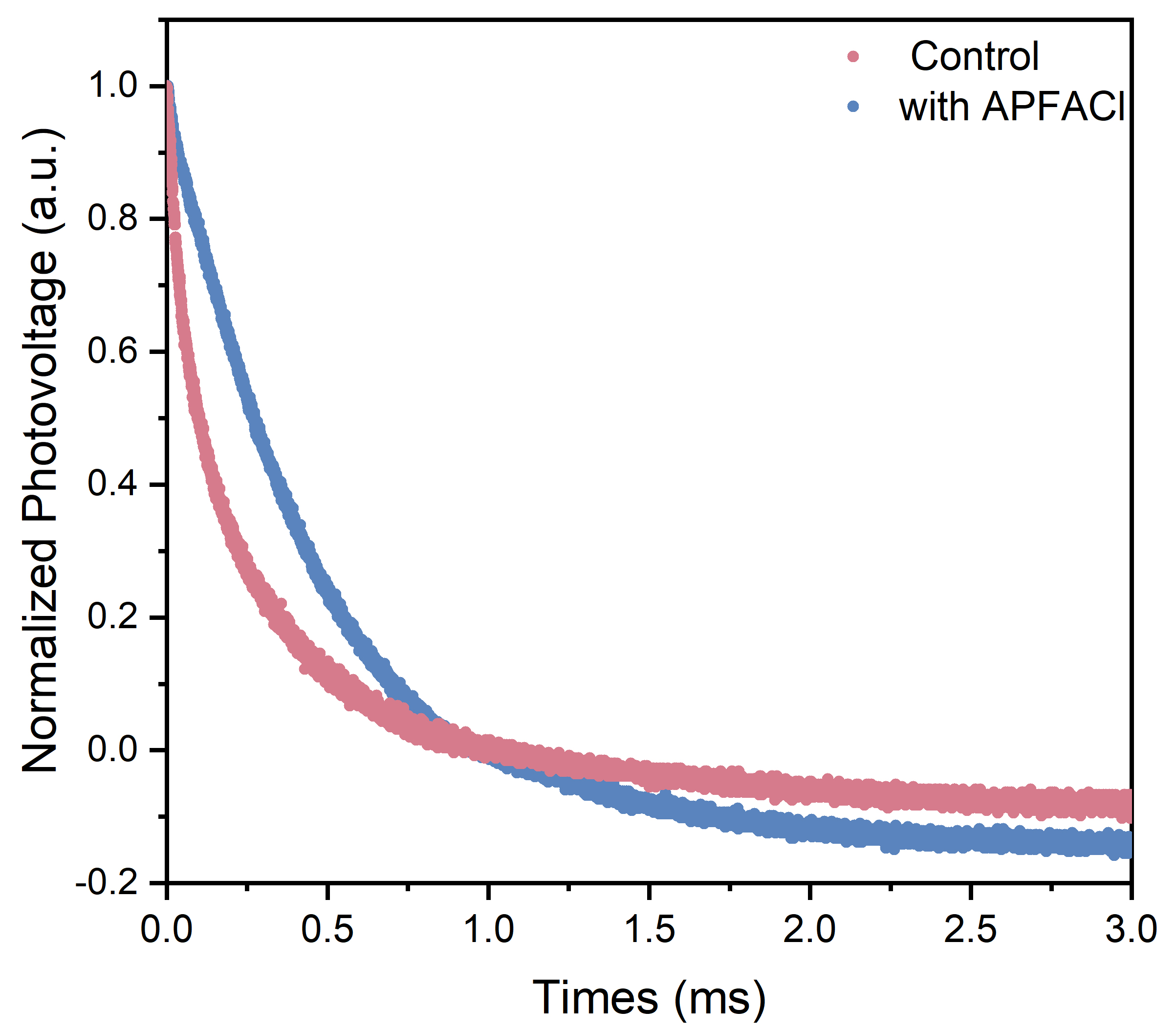


**Figure S17.** Transient photovoltage (TPV) decay curves of control and APFACl-treated devices.


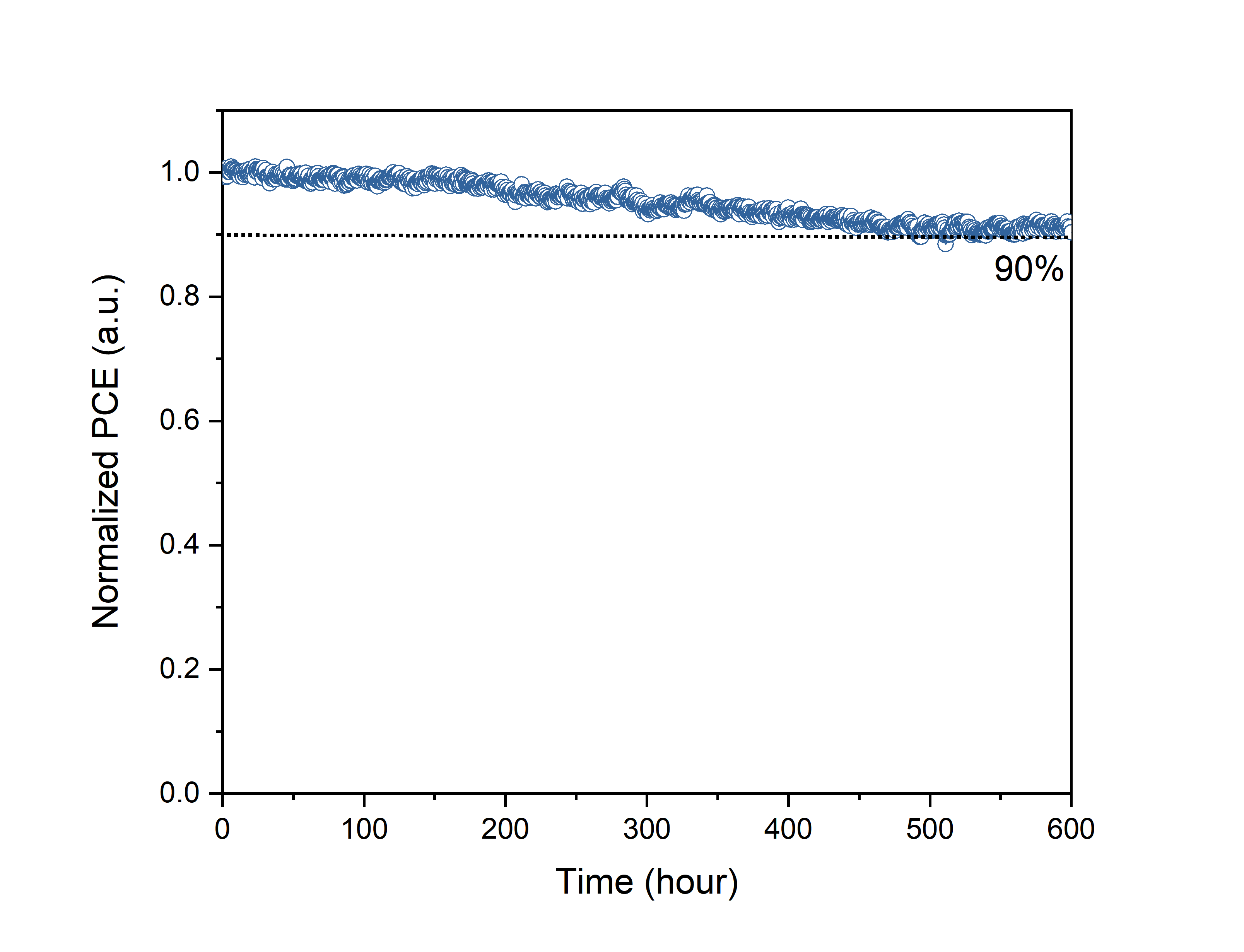


**Figure S18.** MPP tracking of encapsulated all-perovskite TSCs under 1-sun illumination (white LED) in N_2_ at 25°C.
